# Molecular findings from 537 individuals with inherited retinal disease

**DOI:** 10.1136/jmedgenet-2016-103837

**Published:** 2016-05-11

**Authors:** Jamie M Ellingford, Stephanie Barton, Sanjeev Bhaskar, James O'Sullivan, Simon G Williams, Janine A Lamb, Binay Panda, Panagiotis I Sergouniotis, Rachel L Gillespie, Stephen P Daiger, Georgina Hall, Theodora Gale, I Christopher Lloyd, Paul N Bishop, Simon C Ramsden, Graeme C M Black

**Affiliations:** 1 Manchester Centre for Genomic Medicine, Central Manchester University Hospitals NHS Foundation Trust, Manchester Academic Health Sciences Centre, St Mary's Hospital, Manchester, UK; 2 Institute of Human Development, University of Manchester, Manchester, UK; 3 Manchester Royal Eye Hospital, Manchester Academic Health Sciences Centre, Central Manchester University Hospitals NHS Foundation Trust, Manchester, UK; 4 Institute of Population Health, University of Manchester, Manchester, UK; 5 Ganit Labs, Bio-IT Centre, Institute of Bioinformatics and Applied Biotechnology, Bangalore, India; 6 School of Public Health, University of Texas Health Science Center, Houston, Texas, USA

**Keywords:** next-generation sequencing, Molecular genetics, inherited retinal dystrophy, bioinformatics, retinitis pigmentosa

## Abstract

**Background:**

Inherited retinal diseases (IRDs) are a clinically and genetically heterogeneous set of disorders, for which diagnostic second-generation sequencing (next-generation sequencing, NGS) services have been developed worldwide.

**Methods:**

We present the molecular findings of 537 individuals referred to a 105-gene diagnostic NGS test for IRDs. We assess the diagnostic yield, the spectrum of clinical referrals, the variant analysis burden and the genetic heterogeneity of IRD. We retrospectively analyse disease-causing variants, including an assessment of variant frequency in Exome Aggregation Consortium (ExAC).

**Results:**

Individuals were referred from 10 clinically distinct classifications of IRD. Of the 4542 variants clinically analysed, we have reported 402 mutations as a cause or a potential cause of disease in 62 of the 105 genes surveyed. These variants account or likely account for the clinical diagnosis of IRD in 51% of the 537 referred individuals. 144 potentially disease-causing mutations were identified as novel at the time of clinical analysis, and we further demonstrate the segregation of known disease-causing variants among individuals with IRD. We show that clinically analysed variants indicated as rare in dbSNP and the Exome Variant Server remain rare in ExAC, and that genes discovered as a cause of IRD in the post-NGS era are rare causes of IRD in a population of clinically surveyed individuals.

**Conclusions:**

Our findings illustrate the continued powerful utility of custom-gene panel diagnostic NGS tests for IRD in the clinic, but suggest clear future avenues for increasing diagnostic yields.

## Introduction

Inherited retinal diseases (IRDs) are a diverse set of Mendelian disorders that are a major cause of inherited blindness across the world. They are caused by the progressive deterioration or the early loss of cells fundamental for the normal function of the retina,^1^ the component of the eye responsible for converting light energy into electrical signals.

IRDs are clinically heterogeneous and vary widely in their severity, age of onset, pathogenesis, manner of disease progression and inheritance pattern. IRDs distinguishable through clinical and electrophysiological investigation include: rod-cone dystrophy and retinitis pigmentosa (RCD/RP); cone dystrophy (CD); cone-rod dystrophy (CRD); macular dystrophy and Stargardt disease (MD/STGD); early-onset retinal disease and Leber congenital amaurosis (EORD/LCA); congenital stationary night blindness (CSNB); and familial exudative vitreo-retinopathy. Notably, IRD can be a feature of a multisystem disorder such as Usher syndrome, Bardet–Biedl syndrome (BBS), Senior–Loken syndrome and Joubert syndrome.

Decades of research have elucidated the genetic basis of IRD, revealing a suite of mutations in over 200 genes.^2^ The genes associated with IRDs can be involved in a variety of processes and functions,^3^ and their tissue expression can range from exclusive expression in the retina to ubiquitous expression across the body. IRDs are predominantly monogenic but a range of inheritance patterns have been described, including: autosomal-dominant, autosomal-recessive, X-linked, digenic and mitochondrial inheritance.

The identification of the genetic basis of IRD can greatly assist the clinical diagnosis, counselling, treatment and management received on a patient-by-patient basis. Since the inception of second-generation DNA sequencing technologies (commonly referred to as next-generation sequencing, NGS)—techniques permitting the surveillance of genetic variation within multiple genes through a single experiment^4^—many clinical laboratories have adopted NGS as a tool for the diagnosis of rare genetic diseases,^5–7^ including IRD.^8^ ^9^ We previously described a custom-gene panel NGS diagnostic service for individuals with IRD.^10^ This follow-up report outlines the success of this 105 gene diagnostic test for the first 537 individuals with IRD referred from worldwide institutions and describes 131 new mutations in 45 genes as a potential cause of disease.

## Materials and methods

### Patient referrals

Patient referrals were made from worldwide clinical institutions. There were no specific requirements for clinical phenotypic descriptions of IRD before diagnostic genomic testing was undertaken, although the reason for referral was requested. All experiments had been approved by the Greater Manchester West Research Ethics Committee and were performed in a UK Accreditation Service Clinical Pathology Accredited Medical Laboratory.

### Enrichment and sequencing

DNA samples were enriched for the coding regions ±50 bp of 105 genes (see online supplementary table S1) and a specified intronic region of the *CEP290* gene, using an Agilent SureSelect Custom Design target-enrichment kit (Agilent, Santa Clara, California, USA). DNA samples were indexed with a unique paired-end barcode and then subjected to multiplexed high-throughput parallel sequencing using either the ABI SOLiD 5500 platform (n=235; Life Technologies, Carlsbad, California, USA) or the Illumina HiSeq 2000/2500 (n=302; Illumina, San Diego, California, USA), following manufacturer's protocols.

10.1136/jmedgenet-2016-103837.supp1 Supplementary tables

### Variant calling

Sequencing reads were demultiplexed and aligned to the *hg19* reference genome before duplicate read removal and variant calling was performed. For ABI SOLiD sequencing reads, demultiplexing, alignment and variant calling were all performed using Lifescope Genomic Analysis software. For Illumina HiSeq sequencing reads, demultiplexing was performed using CASAVA V.1.8.2. (Illumina), alignment was performed using Burrows-Wheeler Aligner short read (BWA-short V.0.6.2) software,^11^ and variant calling was performed using the UnifiedGenotyper^12^ within the Genome Analysis Tool Kit^13^ (GATK-lite V.2.0.39), after base quality score recalibration and indel realignment. To reduce the number of potential false-positive variants from NGS, we primarily limited the clinical analysis of genomic variants to those with sequencing quality metrics above specific criteria. These metrics were calculated as a result of pilot and control sample analyses, which have been previously published.^10^ ^14^ For the ABI SOLiD, we considered single-nucleotide variants (SNVs) with ≥18× sequencing depth and a minimum mean quality value (MQV) >18, and considered indels with support from >5× independent sequencing reads. For the Illumina HiSeq, we considered SNVs with ≥50× independent sequencing reads and ≥45 MQV, and considered indels with support from >25% of the aligned and independent sequencing reads.

### Variant interpretation

A summary of our strategy to clinically interpret genetic variants is provided in online supplementary table S2. Clinical interpretation was restricted to variants within coding regions ±5 bp of the 105 genes included in online supplementary table S1 and an intronic variant, c.2991+1655A>G, in the *CEP290* gene. We considered variants with a frequency >1% in control population databases (Exome Variant Server, ESP-6500; dbSNP V.135) as benign polymorphisms, providing the cohort size was sufficiently large and diverse, and the sequencing read depth exceeded an average of 18× in the Exome Variant Server (EVS). Variants with a frequency below 1% in SNP databases but with a high recurrence rate in-house were classified as neutral or proven to be NGS run artefacts by Sanger sequencing. The pathogenicity of the remaining genetic variants was determined through extensive appraisal of the variant's predicted consequence (annotations performed against the Ensembl V.68 database), the scientific literature, the patient's clinical referral (genotype–phenotype correlations) and in silico modelling including SIFT^15^ and PolyPhen-2.^16^ These criteria for variant interpretation are consistent with the guidelines recently outlined by the American College of Medical Genetics.^17^

### Clinical decision making

A confirmed molecular diagnosis was provided for individuals with variants determined as ‘clearly pathogenic’ or ‘likely pathogenic’ (see online supplementary table S2) in a disease-causing state (eg, homozygous or compound heterozygous in a gene known to cause recessive IRD). A provisional molecular diagnosis was reported for individuals with ‘variants of unknown clinical significance’ (see online supplementary table S2) that were found in a disease-causing state (eg, compound heterozygous with another ‘clearly pathogenic’ variant in a gene known to cause recessive IRD). If ‘clearly pathogenic’ or ‘likely pathogenic’ variants were identified in more than one gene in a disease-causing state the family history, phenotypic presentation and evidence for variant pathogenicity was discussed in detail within a clinical multidisciplinary team meeting. Variants determined as ‘clearly pathogenic’ or ‘likely pathogenic’ in a carrier state (eg, heterozygous in a gene known to cause recessive IRD) were clinically reported as carrier findings. Cascade testing was offered for relevant family members to assist with the interpretation of variant pathogenicity and to clarify the risks to additional family members.

### Variant validation

We performed PCR and bidirectional capillary sequencing to confirm the zygosity and the presence of disease-causing and carrier variants before they were clinically reported.

### Retrospective variant analysis

All clinically analysed SNVs were also retrospectively compared with the Exome Aggregation Consortium (ExAC) database in October 2015 to determine their frequency within a large and ethnically diverse population data set. Novel and potentially disease-causing variants, absent from the literature at the time of clinical analysis, were retrospectively compared with the Human Gene Mutation Database, *HGMD*,^18^ in October 2015. Mutated genes determined to account for molecular diagnoses were compared with their dates of discovery, available in the RetNet database.^2^

## Results

### Clinical details

#### Demographics and classifications

We performed diagnostic NGS testing for 537 patients (287 males and 250 females) referred with clinical indications of IRD. All of the 537 individuals were initially referred on a singleton basis.

#### Phenotype classification

IRDs have a diverse spectrum of overlapping clinical presentation, which can vary by age of onset, extent of visual impairment, nature of disease progression and involvement of additional clinical features. We grouped all referred patients into 10 distinct clinical classifications (see online supplementary table S3). The most common referral was non-syndromic RCD/RP (n=250). The most common referral of syndromic disease was Usher syndrome (n=38), a disorder characterised by neurosensory hearing loss and visual impairment. There were eight cases of suspected syndromic ciliopathies referred, including seven cases of BBS.

### Molecular results

#### Sequencing statistics

A total of 2 089 243 343 aligned, ontarget and unique sequencing reads were generated by NGS. For the 235 patients analysed through ABI SOLiD sequencing, an average of 6 537 522 aligned, ontarget and unique sequencing reads were generated for each patient. For the 302 patients analysed through Illumina HiSeq sequencing, an average of 1 830 879 aligned, ontarget and unique sequencing reads were generated for each patient. The diagnostic assay achieved an average coverage at ≥20× for 98.4% and at ≥50× for 97.3% of the region clinically analysed for the 537 referred patients (see online supplementary figure S1). Sequencing data generated through the Illumina HiSeq covered significantly more of the clinically analysed region than sequencing data generated through the ABI SOLiD at ≥20× coverage (see online supplementary figure S1; HiSeq=99.0±0.01%, SOLiD=97.6±0.05%, p<0.0001) and ≥50× coverage (see online supplementary figure S1; HiSeq=98.3±0.03%, SOLiD=95.9±0.09%, p<0.0001).

#### Variant analysis burden

A total of 143 675 variants were initially identified in the 537 patients (table 1). Using a bioinformatics pipeline, we filtered the variants for their quality and frequency in control populations. This restricted the number of variants for analysis to 4542, an average of 8.4 variants per referred individual. Most of the clinically analysed variants were single-nucleotide alterations (SNVs), with an average of 8.1 per patient. Heterozygous SNVs provided the largest burden for analysis, with 4172 detected (an average of 7.8 per patient), but only 6% (n=252) reported as a cause of disease after extensive clinical analysis (table 1). There were 2266 unique SNVs clinically analysed, with 574 identified in more than 1 individual. An indel event was clinically analysed, on average, in a third of the referred patients.

**Table 1:** The total number of genetic variants found during the diagnostic screening process

Type	Hom	Het	Hemi	Het–het	Total
Raw calls					
SNVs	46 405	90 981	338	82	137 806
Indels	2182	3653	12	22	5869
Total	48 587	94 634	350	104	143 675
Clinically analysed					
SNVs	166	4172	20	0	4358
Indels	27	150	7	0	184
Total	193	4322	27	0	4542
Clinically reported					
SNVs	58	252	9	0	319
Indels	31	48	4	0	83
Total	89	300	13	0	402

The zygosity of *raw calls* and *clinically analysed* variants is estimated from the sequencing read pileups of next-generation sequencing (NGS) data. The zygosity of *clinically reported* variants is confirmed through an alternative technique. *hom*, homozygous variants; *het*, heterozygous variants, *hemi*, hemizygous variants found on chrX in males; *het–het*, variants with two unique alternative alleles differing from the reference allele (*hg19*).

**Figure 1:**
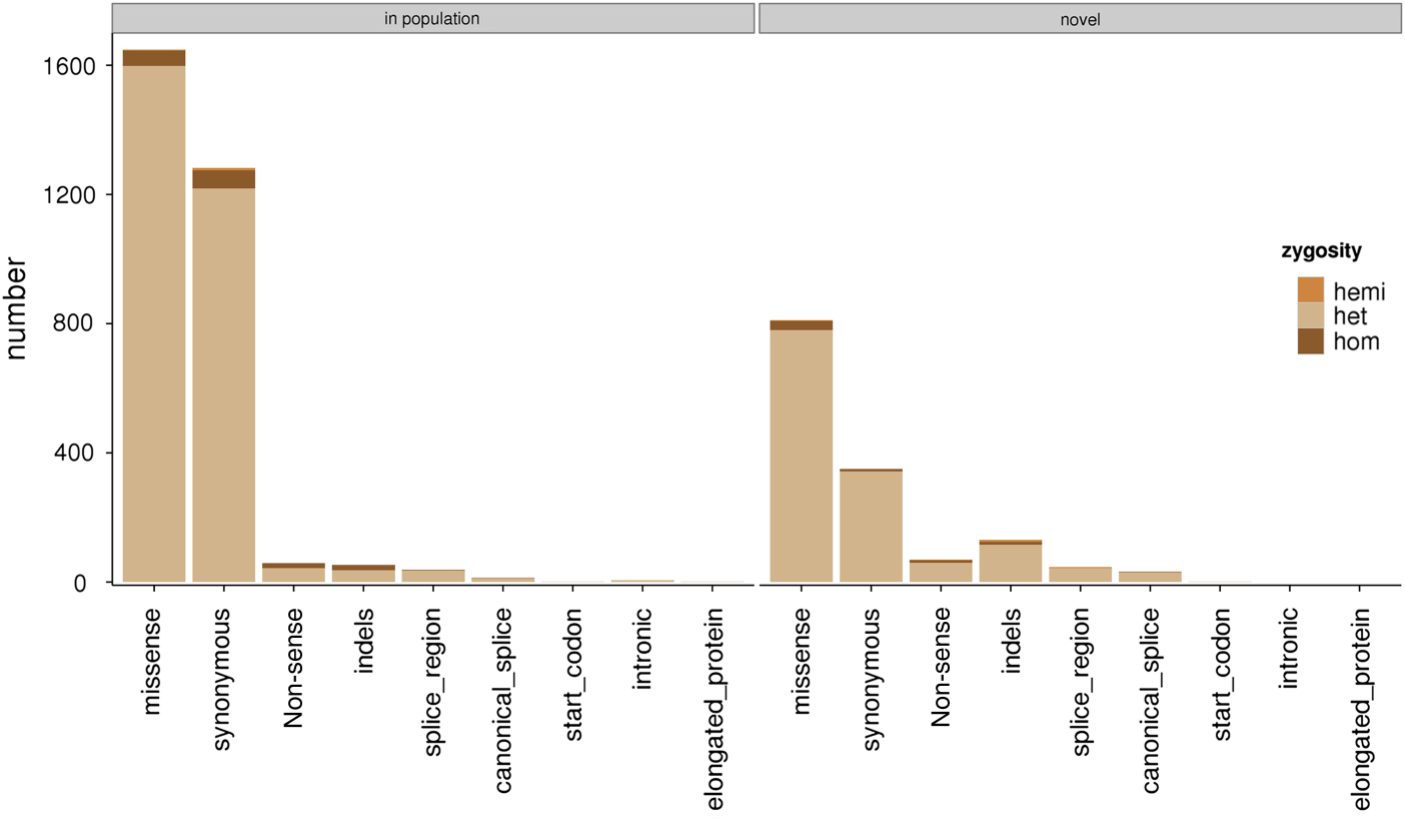
The variant analysis burden. The numbers of variants analysed by clinically accredited scientists by their expected consequences on the encoded protein and frequency in control populations. Annotations are performed against the specified transcripts in online supplementary table S1 using V.68 of the Ensembl database and population frequencies available in dbSNP and EVS.

At the time of analysis, 30% of SNVs (1311/4358) and 71% of indels (131/184) were neither present in dbSNP or in EVS. This included 811 missense variants, 70 nonsense variants, 105 coding region indel events, 26 splice region indel events, 350 synonymous variants, 2 start codon variants, 32 canonical splice site variants and 46 splice region variants (novel; figure 1). We clinically analysed 135 deletion events (105 heterozygous and 30 homozygous), 48 insertion events (46 heterozygous and 2 homozygous) and 1 heterozygous deletion–insertion event. Eighty percent (147/184) of the indel events were present in the coding regions of genes, and 110 of these were expected to cause premature termination of the protein product (84 heterozygous and 26 homozygous). A small number of clinically analysed variants were determined as false positives after analysis through PCR and bidirectional capillary sequencing techniques (see online supplementary table S4).

#### Clinical decision making

We identified a molecular diagnosis for 271 of the 537 referred patients (51%). The molecular findings underpinning these diagnoses are reported in online supplementary table S5. We have provided a molecular diagnosis for recessive IRD in 208 cases (homozygous, n=88; compound heterozygous, n=120), dominant IRD in 50 cases and X-linked IRD in 13 cases. We found no clear instances of digenic or polygenic inheritance. We informed clinicians of 154 individuals who carry ‘clearly pathogenic’ or ‘likely pathogenic’ variants in a carrier state, that is, heterozygous in a gene known to underpin disease inherited in a recessive manner, 59 of whom received a molecular diagnosis after the identification of ‘clearly pathogenic’ or ‘likely pathogenic’ variants in other disease-causing genes (dominant IRD, n=11; recessive IRD, n=48). Eighty families were referred for cascade testing after receiving molecular results from NGS testing for the referred proband. We confirmed compound heterozygous variants to be *in-trans* in 36 cases, confirmed homozygosity in 11 cases, identified de novo variants in 3 cases and identified 16 cases of additional variant segregation with IRD. In a single case, we identified heterozygous variants underpinning a provisional molecular diagnosis to be *in-cis*, altering the counselling that was received by the referred individual.

Several individuals were found to have ‘clearly pathogenic’ or ‘likely pathogenic’ variants in a disease-causing state that would not be expected to cause the IRD subtype declared by the referring clinician (see online supplementary table S6). For example, two individuals referred with clinical indications of RP were found to have ‘likely pathogenic’ mutations in the *CHM* gene, a cause of choroideremia. These genomic findings led to the re-evaluation of initial clinical descriptions, and in some cases refined or altered the initial clinical diagnosis. In a single case (13005797; online supplementary table S5), bidirectional capillary sequencing targeted at a region of *GUCY2D*, which had been poorly covered through NGS (460 nucleotides of a coding exon with <50× coverage) identified a novel heterozygous missense variant, c.380C>T p.(Pro127Arg), that had not been identified through diagnostic NGS. This large protein-coding exon of *GUCY2D* (chr17:7906361–7907091) has a GC content of 76.20%. Homozygous and compound heterozygous mutations in *GUCY2D* are known to cause EORD/LCA.^19^ When considered in combination with a heterozygous frameshift mutation in *GUCY2D*, c.2595delG, the identified missense variant was concluded to likely account for a molecular diagnosis of autosomal-recessive EORD/LCA.

#### Genetic heterogeneity

In total, 402 genetic variants are reported to account for a molecular diagnosis of IRD (273 variants with a single occurrence and 38 variants reported multiple times). Variants reported in multiple individuals ranged from presence in 2 (n=22) to 11 patients (n=1). The most commonly reported variant is *CERKL* c.375C>G p.(Cys125Trp), which is reported to coincide with clinical presentations of IRD from four disease subgroups: one case of CD; three cases of CRD; four cases of RCD/RP and three cases of MD/STGD.

**Figure 2:**
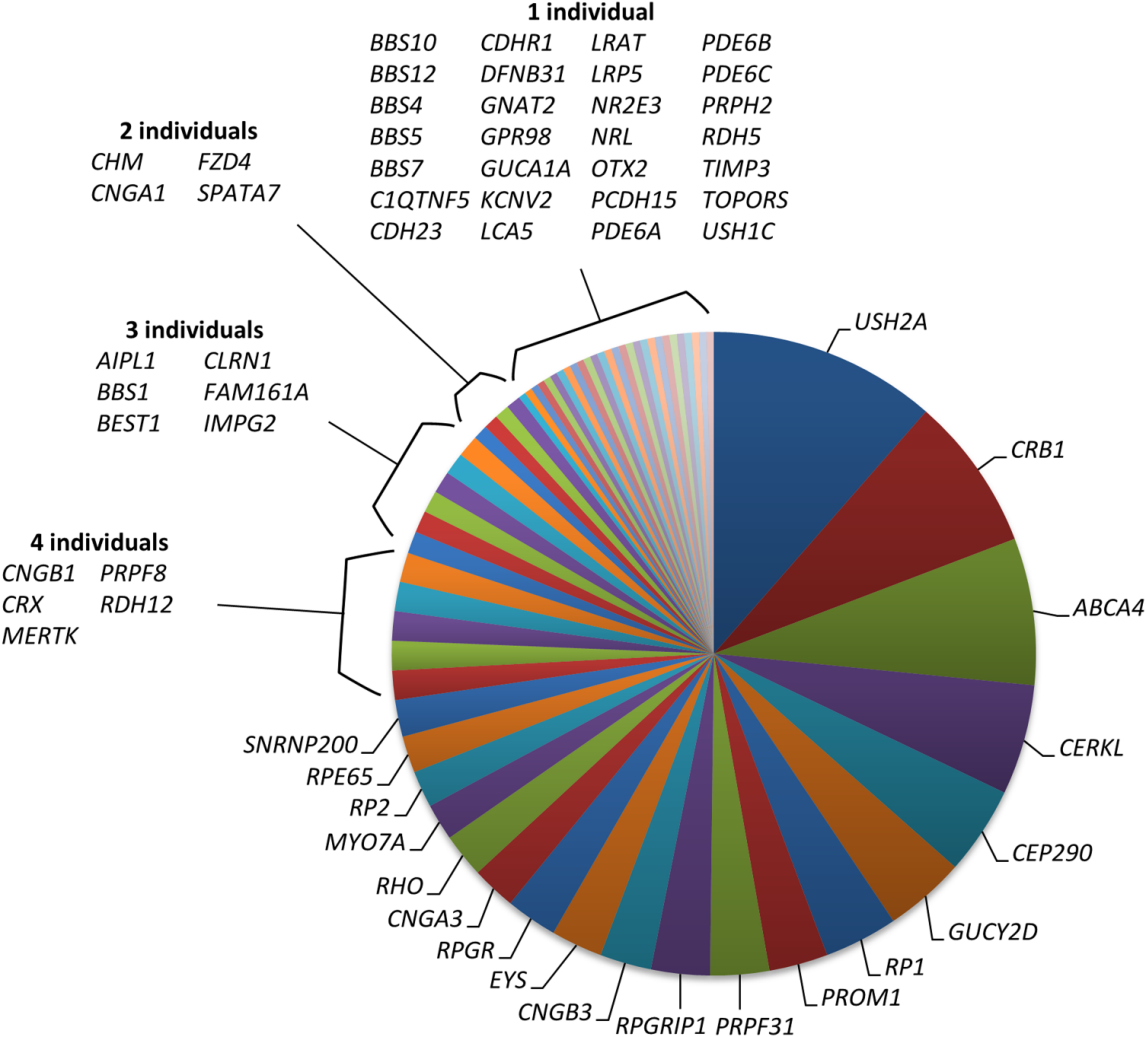
The genetic basis of inherited retinal disease in 271 individuals with a confirmed or provisional molecular diagnosis. Each segment illustrates the number of individuals with genetic variants determined as a cause of inherited retinal disease.

Variants accounting for a molecular diagnosis of IRD were found in 62 of the 105 genes included in the diagnostic NGS assay (figure 2). Most of the genes with disease-causing variants are restricted to a single disease referral category (see online supplementary table S7; 66%, n=41), the most frequent of which are mutations causing recessively inherited RCD/RP in the *EYS* gene (n patients=7) and dominantly inherited RCD/RP in the *RHO* gene (n patients=6). The remaining 21 genes are reported with pathogenic variants in patients referred from two or more disease referral categories (see online supplementary table S7). The gene associated with the most highly variable phenotype is *ABCA4*, which was concluded to contain pathogenic variants causing IRD in 23 patients referred from five discrete referral categories, including: 12 patients with MD/STGD; 5 with CRD; 4 with RCD/RP and 1 individual with CD. Disruption of the *ABCA4* gene was also concluded to be the cause of visual impairment in a single patient referred with Usher syndrome but is not expected to contribute to the clinical presentation of hearing loss.

#### Mutation consequences

We determined 311 unique mutations to be the cause of disease. These included 137 missense variants, 20 canonical splice site variants, 76 nonsense variants, 54 out-of-frame insertion or deletion events, 1 variant disrupting the start codon, 6 synonymous variants, 9 splice region variants, 1 intronic variant and 7 inframe deletions. Fifty-two percent (162/311) of the variants reported to account for a molecular diagnosis were neither present in dbSNP or EVS. Specifically, 71% (97/137) of the clinically reported missense variants, 55% (42/76) of clinically reported nonsense SNVs and 63% (34/54) of clinically reported out-of-frame indel events were neither present in dbSNP or EVS.

Of the 137 clinically reported missense variants, 30 had functional support of their disruption to the encoded protein, 49 had disease segregation evidence in the literature without functional support, 6 had evidence for an alternative amino acid substitution at the same residue as a cause of disease and 52 were determined as a novel cause of disease (see online supplementary table S8). One hundred and thirty clinically reported variants are expected to cause premature termination during protein synthesis, 76 of these are SNVs and 54 are out-of-frame small insertion or deletion events. Fifty-one percent (66/130) of these loss-of-protein-function events were determined as novel causes of disease; this included 35 nonsense SNVs and 31 small indels (see online supplementary table S8).

#### Retrospective variant analysis

The average allele frequency in ExAC of the 2266 unique clinically analysed SNVs was 0.12; however, 12% (517/4358) of all clinically analysed SNVs were found to be at a greater frequency than 1% in the ExAC database. The average allele frequency in ExAC of variants clinically reported as a cause of disease was 0.02 (median=0 and max=0.78).

One hundred and forty-four variants were determined as novel causes of disease at the time of clinical analysis. Ninety-one percent (131/144) of these variants remained novel causes of disease after comparison with the HGMD in October 2015. This included 46 missense variants, 12 canonical splice site variants, 33 nonsense variants, 28 out-of-frame insertion or deletion events, 4 synonymous variants, 5 splice region variants and 3 inframe deletions. All of these variants are reported in online supplementary table S8 and have been submitted to the ClinVar database.

**Figure 3:**
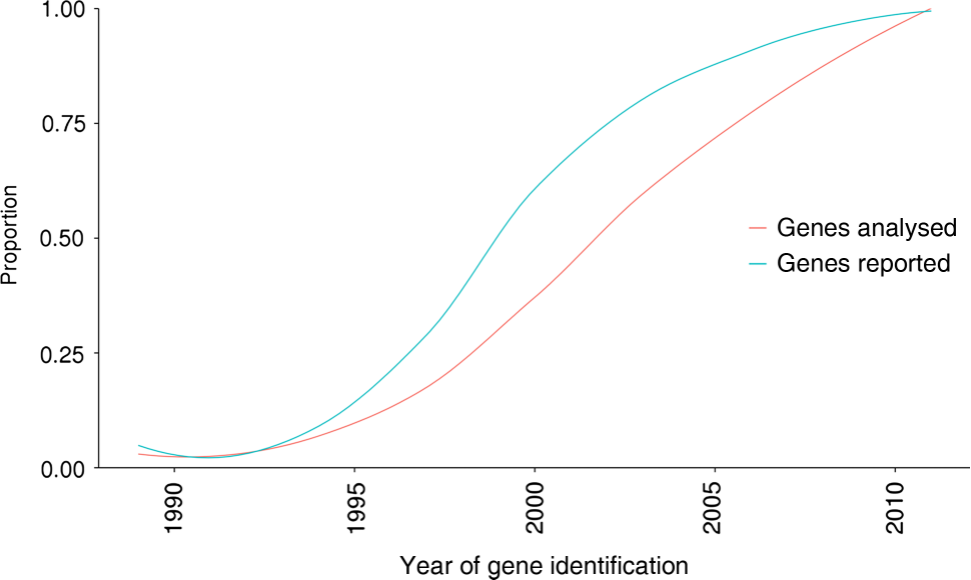
The relationship between the genes accounting for molecular diagnoses and the year of their discovery. The curves illustrate the trends in the proportion of 271 molecular diagnoses accounted for by the analysis of 105 genes through diagnostic next-generation sequencing.

Sixty-five of the 105 genes surveyed (62%) were identified between 1995 and 2004, and mutations in these genes accounted for a molecular diagnosis in 214 of the 271 cases (79%; figure 3). This equates to a rate of 3.3 diagnoses per gene analysed. In contrast, the 33 genes included in our analysis that were identified post-2005 only accounted for 41 of the 271 molecular diagnoses (15%; figure 3), a rate of 1.2 diagnoses per gene analysed.

## Discussion

Developments in DNA sequencing technology have enabled the transition from single gene and known mutation diagnostic assays to comprehensive surveys of variation within all genes known to cause disease phenotypes. This has shifted the diagnostics bottleneck from the detection of disease-causing variation to the clinical interpretation of the plethora of genetic variants identified. Here, we discuss the molecular findings from a NGS diagnostic test that has been employed in a clinical context to survey genes known to be associated with IRD, a clinically and genetically heterogeneous set of Mendelian disorders that affect millions of individuals worldwide.^20^ The findings derived from a large cohort of individuals referred specifically for diagnostic genomic testing.

We have clinically reported 402 mutations as a cause or a potential cause of disease in 62 of the 105 genes surveyed. These variants account or likely account for the clinical diagnosis of IRD in 51% of the 537 individuals referred for diagnostic testing, including 208 autosomal-recessive, 50 autosomal-dominant and 13 X-linked recessive cases. Other studies employing NGS have shown higher molecular diagnostic rates than reported in this study, including Eisenberger and colleagues,^21^ Zhao and colleagues^22^ and the Saudi Mendeliome group.^23^ We attribute differences in diagnostic success rates to a number of factors. First, the diagnostic service is provisioned for worldwide referral institutions on a singleton basis and there are no prespecified criteria for the clinical phenotyping of referred individuals. Detailed clinical phenotyping in complement to genomic analysis has been shown to discover and accelerate diagnoses in the clinic.^24^ ^25^ Second, the 105 gene enrichment does not include all genes that are now known as a cause of IRD, and application of whole exome sequencing (WES) or whole genome sequencing (WGS) techniques to individuals without a molecular diagnosis has identified variants in other genes as a cause of disease, for example, mutations in *IQCB1* identified through WES.^26^ It is of note that we have recently expanded the diagnostic service to include an additional 71 genes and pathogenic intronic variants associated with IRD. This expansion intends to meet the clinical need for the surveillance of genes known to underpin CSNB. Third, there were technical limitations with early versions of the diagnostic service, for example, insufficient coverage achieved by the ABI SOLiD sequencing platform over all 288 087 DNA bases which are clinically surveyed (see online supplementary figure S1).

The use of NGS in the clinic has drastically increased the diagnostic yields routinely achieved for IRD. However, there still remains an inability to clinically survey the final AG-rich exon of *RPGR* (orf15) through the NGS techniques described in this study and by others. Mutations in *RPGR*orf15 are well documented as a major cause of X-linked RP,^27^ but bidirectional capillary sequencing techniques remain the gold standard clinical screening tool for this region of the genome.^28^ Complementing diagnostic NGS services with bidirectional capillary sequencing of regions which remain elusive to NGS will increase diagnostic yields. Moreover, genetic variant detection in clinical diagnostics requires clinically validated bioinformatics techniques. Such techniques have evolved with the clinical requirements of diagnostic services. Validation of bioinformatics techniques to detect complex insertion and deletion events, for example, Pindel,^29^ and complete exon deletions and duplications, for example, ExomeDepth,^30^ will undoubtedly increase the diagnostic yields described in this study, and in others. Finally, the expansion of analyses to non-coding and regulatory regions of known disease-causing genes will increase diagnostic yields for IRD. We observed an interesting trend in our cohort of predominantly Western European and South Asian individuals, which suggests that newly identified genes are becoming increasingly rarer causes of disease (figure 3). The striking observation that 79% of cases with molecular diagnoses are accounted for by mutations in genes discovered between 1995 and 2004 suggests that while the continued addition of new disease-causing genes will of course improve diagnostic services, the comprehensive evaluation of protein-coding and regulatory variants affecting known disease-causing genes may have a greater influence on diagnostic yield. For example, the genomic sequencing of genes known to cause IRD has identified large deletions,^14^ deeply intronic disease-causing mutations^31^ and variants in regulatory regions,^32^ which would elude traditional custom panel NGS analysis.

A significant complication of implementing NGS as a diagnostic service is the number of variants, which require clinical interpretation on a patient-by-patient basis (table 1), many of which have not previously been defined in control populations (figure 1). However, a clear advantage of NGS diagnostic approaches over other high-throughput techniques is the capability to detect novel disease-causing variation,^33^ defined as variants that have not previously been reported as disease causing in the literature or in the HGMD. We have reported 144 novel disease-causing variants as a possible cause of IRD (see online supplementary table S8). Thirteen of these variants determined as novel at the time of clinical analysis have since been described as disease causing in other studies, for example, *USH2A* c.11713C>T p.(Arg3905Cys)^34^ and *PRPF31* c.1060C>T p.(Arg354Ter).^35^ Many of the reported novel variants are expected to cause disruption of normal translation, including 35 nonsense variants, 6 disruptions of canonical splice sites and 31 out-of-frame coding indels. Most novel variants are clinically reported on a single occasion; however, some are reported to cause disease in multiple individuals, for example, *CERKL* c.193G>T p.(Glu65Ter) in five individuals and *EYS* c.1155T>A p.(Cys385Ter) in three individuals. None of the novel missense variants, splice region variants or inframe indels reported in this study are determined to cause disease on multiple occasions, and further disease segregation and/or functional study will greatly assist their clinical interpretation.

In summary, we illustrate the powerful clinical utility of a diagnostic NGS test for individuals with inherited visual impairment. We add to evidence of known pathogenic mutations through further correlation in individuals who present with clinical indications of IRD (see online supplementary table S5) and outline 131 novel disease-causing variants that are not previously described in individuals with IRD.
