# Supplementary material for: Molecular findings from 537 individuals with inherited retinal disease
Source: J Med Genet. 2016 May 11;53(11):761–7. doi: 10.1136/jmedgenet-2016-103837 (PMC5106339; doi:10.1136/jmedgenet-2016-103837)
Supplement: Supplementary tables [file jmedgenet-2016-103837supp_tables.pdf]

Supp. Table 1. Specified transcripts for 105 gene symbols analysed by targeted NGS diagnostic testing.

HGNC symbol	RefSeq Transcript ID	HGNC symbol	RefSeq Transcript ID	HGNC symbol	RefSeq Transcript ID
ABCA4	NM_000350.2	PRPF31	NM_015629.3	IMPG2	NM_016247.3
ADAM9	NM_003816.2	PRPF6	NM_012469.3	KCNV2	NM_133497.3
AIPL1	NM_014336.3	PRPF8	NM_006445.3	KLHL7	NM_001031710.2
ARL6	NM_032146.3	PRPH2	NM_000322.4	IMPG2	NM_016247.3
BBS1	NM_024649.4	RAX2	NM_032753.3	KCNV2	NM_133497.3
BBS10	NM_024685.3	RBP3	NM_002900.2	KLHL7	NM_001031710.2
BBS12	NM_001178007.1	RD3	NM_183059.2	LCA5	NM_181714.3
BBS2	NM_031885.3	RDH12	NM_152443.2	LRAT	NM_004744.3
BBS4	NM_033028.3	RDH5	NM_001199771.1	LRP5	NM_002335.2
BBS5	NM_152384.2	RGR	NM_002921.3	MERTK	NM_006343.2
BBS7	NM_176824.2	RGS9	NM_001165933.1	MKKS	NM_018848.2
BBS7	NM_018190.3	RGS9	NM_003835.3	MKS1	NM_001165927.1
BBS9	NM_198428.2	RHO	NM_000539.3	MKS1	NM_017777.3
BEST1	NM_004183.3	RIMS1	NM_014989.4	MYO7A	NM_000260.3
C1QTNF5	NM_015645.3	RIMS1	NM_001168407.1	NDP	NM_000266.3
C2orf71	NM_001029883.1	CRB1	NM_201253.2	RIMS1	NM_001168410.1
CA4	NM_000717.3	CRX	NM_000554.4	RLBP1	NM_000326.4
CACNA2D4	NM_172364.4	DFNB31	NM_015404.3	ROM1	NM_000327.3
CDH23	NM_022124.5	DHDDS	NM_024887.2	RP1	NM_006269.1
CDHR1	NM_001171971.1	EFEMP1	NM_001039348.2	RP1L1**	NM_178857.5
CDHR1	NM_033100.2	ELOVL4	NM_022726.3	RP2	NM_006915.2
CEP290*	NM_025114.3	EYS	NM_001142800.1	RP9	NM_203288.1
CERKL	NM_001030311.2	FAM161A	NM_001201543.1	RPE65	NM_000329.2
CHM	NM_000390.2	FSCN2	NM_001077182.2	RPGR***	NM_001034853.1
CLRN1	NM_052995.2	FZD4	NM_012193.3	RPGRIP1	NM_020366.3
CLRN1	NM_001195794.1	GNAT2	NM_005272.3	RS1	NM_000330.3
CNGA1	NM_001142564.1	GPR98	NM_032119.3	SAG	NM_000541.4
CNGA3	NM_001298.2	GUCA1A	NM_000409.3	SEMA4A	NM_022367.3
CNGB1	NM_001297.4	GUCA1B	NM_002098.5	SNRNP200	NM_014014.4
CNGB3	NM_019098.4	GUCY2D	NM_000180.3	SPATA7	NM_018418.4
NR2E3	NM_014249.2	IDH3B	NM_006899.2	TEAD1	NM_021961.5
NRL	NM_006177.3	IDH3B	NM_174855.1	TIMP3	NM_000362.4
OTX2	NM_021728.2	IMPDH1	NM_000883.3	TOPORS	NM_005802.4
PCDH15	NM_001142763.1	IMPG2	NM_016247.3	TRIM32	NM_012210.3
PCDH15	NM_001142769.1	KCNV2	NM_133497.3	TTC8	NM_144596.2
PCDH15	NM_001142771.1	KLHL7	NM_001031710.2	TULP1	NM_003322.3
PCDH15	NM_001142770.1	LCA5	NM_181714.3	UNC119	NM_005148.3
PDE6A	NM_000440.2	LRAT	NM_004744.3	UNC119	NM_054035.2
PDE6B	NM_000283.3	LRP5	NM_002335.2	USH1C	NM_005709.3
PDE6C	NM_006204.3	MERTK	NM_006343.2	USH1C	NM_153676.3
PDE6G	NM_002602.3	MKKS	NM_018848.2	USH1G	NM_173477.2
PITPNM3	NM_031220.3	MKS1	NM_001165927.1	USH2A	NM_206933.2
PRCD	NM_001077620.2	MKS1	NM_017777.3	ZNF513	NM_144631.5
PROM1	NM_006017.2	MYO7A	NM_000260.3		
PRPF3	NM_004698.2	NDP	NM_000266.3		

* Testing of the common intron 26 mutation c.2991+1655A>G in CEP290 is included in this analysis
** Analysis of the coding region of exon 4 of the RP1L1 gene is not included
*** Analysis of the coding region of the final exon (*orf15*) of *RPGR* it is not included *HGNC*, gene symbols approved by the HUGO Gene Nomenclature Committee[1]

1. Gray KA, Yates B, Seal RL, Wright MW, Bruford EA. Genenames.org: the HGNC resources in 2015. Nucleic Acids Res 2015;43:D1079-85.

Supp Table 2. Mutation classification criteria.

1 Clearly not pathogenic

- Variant has a frequency >1% in either EVS or dbSNP
- Variant is not present in HGMD
- Variant has no evidence for its pathogenesis

2 Unlikely to be pathogenic

- Variant has a frequency <1% in EVS and <1% in dbSNP,
- Variant is not present in HGMD
- Variant does not have evidence for its pathogenicity (*in-silico*, functional or segregation)

3 Variant of unknown significance

- Variant has a frequency <1% in EVS and <1% in dbSNP,
- Variant is not present in HGMD
- Variant does not have clear evidence for its pathogenicity (functional or segregation)
- Variant has strong and multiple lines of *in-silico* evidence to disrupt protein structure and/or normal translation

(a) Variant is not clinically reported

- Variant is not found in a *disease-causing* state

(b) Variant is clinically reported

- Variant is found in a *disease-causing* state (e.g. homozygous or with another ‘clearly pathogenic’ heterozygous variant),
- Variant may be found in a gene highly specific to the patient’s clinical presentation of IRD,

4 Likely pathogenic

- Variant has a frequency <1% in EVS and <1% in dbSNP,
- Variant may/may not be in HGMD,
- Variant may have been shown to segregate with disease previously in a single family,
- Variant does not have functional evidence for its pathogenicity,
- Variant has strong and multiple lines of *in-silico* evidence to disrupt protein structure and/or normal translation,
- Variant may be in a hotspot for known pathogenic mutations within a gene,
- Variant may be in a gene highly specific to the patient’s clinical presentation of IRD,

5 Clearly pathogenic

- Variant has a frequency <1% in EVS and <1% in dbSNP,
- Variant is present in HGMD,
- Variant may have been shown to segregate with disease previously in multiple families,
- Variant may have functional evidence for its pathogenicity,
- Variant has strong and multiple lines of *in-silico* evidence to disrupt protein structure and/or normal translation

Supp Table 3. Clinical indications of individuals referred with inherited retinal disease for diagnostic genomic testing.

Clinical diagnosis	Number of referred cases
Retinitis pigmentosa **or** ***rod-cone dystrophy*** **(RP/RCD)**	250
Leber congenital amaurosis **or** ***early onset rod-cone dystrophy*** **(EORD/LCA)**	78
Other(indication not included in this list, or not defined)	42
Stargardt disease **or** ***macular dystrophy*** **(STGD/MD)**	46
Usher syndrome	38
Cone-rod dystrophy **(CRD)**	39
Achromotopsia **or** ***cone dystrophy*** **(CD)**	27
Suspected syndromic ciliopathy	8
Familial Exudative Vitreoretinopathy **(FEVR)**	5
Choroideremia **(CHM)**	4

Supp. Table S4. Recurrent single nucleotide variants identified by NGS which were determined as false positives after PCR amplification and bidirectional capillary sequencing.

Variant	false positive validations
BBS7 c.166-5T>C	10
BBS7 c.171G>T	9
BBS7 c.181T>A	8
USH2A c.10246T>G	4
ABCA4 c.1356+4A>G	3
RBP3 c.1652A>C	6
RP1L1 c.3981A>G	3
CEP290 c.4006A>T	2
ABCA4 c.4530G>C	6

Supp. Table S5. Genetic variants reported to referring clinicians, including causative, likely causative, and variants of unknown clinical significance.

MCGM ID	Genomic loci *hg19*	Variant details *specified transcripts, Table S1*	Zygosity	Supporting evidence from literature	
				Disease segregation	Functional
**Patients referred with CD**					
**12004242**	chr8:87656009-87656009	CNGB3 c.1148delC	hom	[1, 2]	-
**13017395**	chr2:182468670-182468670	CERKL c.375C>G p.(Cys125Trp)	hom	[3, 4]	[5]
**13015689**	chr2:182413512-182413513	CERKL c.1045_1046delAT	hom	novel	-
**13003908**	chr2:99012481-99012481	CNGA3 c.848G>A p.(Arg283Gln)	hom	-	[6]
**13010246**	chr17:7918018-7918018	GUCY2D c.2512C>T p.(Arg838Cys)	het	[7, 8]	[9]
**13011432**	chr17:7918019-7918019	GUCY2D c.2513G>A p.(Arg838His)	het	[8]	[9]
**13010483**	chr1:94473287-94473287<br>chr1:94471065-94471065	ABCA4 c.5908C>T p.(Leu1970Phe)<br>ABCA4 c.6079C>T p.(Leu2027Phe)	het<br>het	[10, 11]<br>[12]	[12]<br>[13]
**13002658**	chr8:87656009-87656009	CNGB3 c.1148delC	hom	[1, 2]	-
**13009042**	chr2:99013274-99013274	CNGA3 c.1641C>A p.(Phe547Leu)	hom	[14, 15]	[16]
**13012218**	chr2:99013190-99013190<br>chr2:99012912-99012912	CNGA3 c.1557G>A p.(Met519Ile)<br>CNGA3 c.1279C>T p.(Arg427Cys)	het<br>het	novel<br>[17-20]	-<br>[18]
**13000861**	chr8:87656009-87656009	CNGB3 c.1148delC	hom	[1, 2]	-
**13017391**	chr17:7918821-7918821	GUCY2D c.2944+1delG	hom	[21-23]	-
**13007934**[+]	chr1:110146104-110146104	GNAT2 c.937C>T p.(Arg313Ter)	hom	[24]	-
**13002676**	chr8:87656009-87656009	CNGB3 c.1148delC	hom	[1, 2]	-
**13015850**	chr8:87656009-87656009	CNGB3 c.1148delC	hom	[1, 2]	-
**12006121**	chr8:87656009-87656009	CNGB3 c.1148delC	hom	[1, 2]	-
**12001563**	chr8:87616320-87616320	CNGB3 c.1781+1G>C	het	[2]	

Supp. Table S5. Genetic variants reported to referring clinicians, including causative, likely causative, and variants of unknown clinical significance.

MCGM ID	Genomic loci *hg19*	Variant details *specified transcripts, Table S1*	Zygosity	Supporting evidence from literature	
				Disease segregation	Functional
	chr8:87656009-87656009	CNGB3 c.1148delC	het	[1, 2]	
**13001685**	chr2:99012912-99012912	CNGA3 c.1279C>T p.(Arg427Cys)	het	[17-20]	[18]
	chr2:99008427-99008427	CNGA3 c.667C>T p.(Arg223Trp)	het	[17, 25]	
**12003493**	chr10:95405761-95405761	PDE6C c.1892A>G p.(Glu631Gly)	het	novel	-
	chr10:95405763-95405763	PDE6C c.1894A>T p.(Arg632Trp)	het	novel	-
**Patients referred with BBS**					
**12014789**	chr4:122749847-122749848	BBS7 c.1712_1713delinsAGA p.(Ser571Ter)	hom	novel	-
**12003699**+	chr11:66293652-66293652	BBS1 c.1169T>G p.(Met390Arg)	hom	[26-29]	[30]
**13013491**+	chr11:66293652-66293652	BBS1 c.1169T>G p.(Met390Arg)	hom	[26-29]	[30]
**13000497**+	chr11:66293652-66293652	BBS1 c.1169T>G p.(Met390Arg)	hom	[26-29]	[30]
**10001098**+	chr4:123664110-123664110	BBS12 c.1063C>T p.(Arg355Ter)	hom	[31]	-
**12009772**	chr15:73023645-73023645	BBS4 c.712-1G>A	hom	novel	-
**13000378**	chr12:76741493-76741494	BBS10 c.271dupT	hom	[32]	-
**Patients referred with CRD**					
**12014625**+	chr1:197403836-197403836	CRB1 c.2843G>A p.(Cys948Tyr)	het	[33-37]	mutation hotspot [34]
	chr1:197396689-197396689	CRB1 c.2234C>T p.(Thr745Met)	het	[33, 35]	mutation hotspot [34]
**13011267**	chr2:182468670-182468670	CERKL c.375C>G p.(Cys125Trp)	het	[3, 4]	[5]
	chr2:182521541-182521541	CERKL c.193G>T p.(Glu65Ter)	het	novel	-
**13005842**+	chr1:197325978-197325978	CRB1 c.1006T>C p.(Cys336Arg)	het	novel	-

Supp. Table S5. Genetic variants reported to referring clinicians, including causative, likely causative, and variants of unknown clinical significance.

MCGM ID	Genomic loci *hg19*	Variant details *specified transcripts, Table S1*	Zygosity	Supporting evidence from literature	
				Disease segregation	Functional
	chr1:197396584-197396584	CRB1 c.2129A>T p.(Glu710Val)	het	[33, 38, 39]	-
**12011000**	chr19:54621779-54621779	PRPF31 c.121C>G p.(Leu41Val)	het	novel	-
**13007236**	chr1:94485164-94485164	ABCA4 c.5170T>C p.(Trp1724Arg)	hom	p.Trp1724Cys [40, 41]	-
**13010981**+	chr19:48339520-48339520	CRX c.121C>T p.(Arg41Trp)	het	[42]	[42, 43]
**11011507**	chr1:94466422-94466422	ABCA4 c.6449G>A p.(Cys2150Tyr)	het	[44, 45]	-
	chr1:94568687-94568687	ABCA4 c.454C>T p.(Arg152Ter)	het	[46]	-
**88301**	chr4:15985950-15985950	PROM1 c.2309delC	het	novel	-
	chr4:16008260-16008261	PROM1 c.1354dupT	het	[47]	-
**82863**+	chr1:94508984-94508984	ABCA4 c.3098delA	het	novel	-
	chr1:94496666-94496666	ABCA4 c.4139C>T p.(Pro1380Leu)	het	[12, 45, 48, 49]	[50]
**990697**+	chr1:94473278-94473278	ABCA4 c.5917delG	het	[51]	-
	chr1:94463488-94463488	ABCA4 c.6658C>T p.(Gln2220Ter)	het	[52-54]	-
**12014678**+	chr12:88476853-88476854	CEP290 c.4966_4967delGA	het	[55]	-
	chr12:88465636-88465636	CEP290 c.5777G>C p.(Arg1926Pro)	het	[56]	-
**11011667**+	chr2:99013321-99013321	CNGA3 c.1688G>A p.(Arg563His)	het	[17]	[57]
	chr2:98986505-98986505	CNGA3 c.67C>T p.(Arg23Ter)	het	[25]	
**13012292**	chrX:38158220-38158220	RPGR c.1234C>T p.(Arg412Ter)	het	[58]	-
**13016156**+	chr2:182468670-182468670	CERKL c.375C>G p.(Cys125Trp)	het	[3, 4]	[5]
	chr2:182423344-182423344	CERKL c.847C>T p.(Arg283Ter)	het	[3]	
**12013927**	chr4:16014893-16014893	PROM1 c.1141+5G>C	hom	novel	-
**12009616**+	chr4:16008260-16008261	PROM1 c.1354dupT	het	[47]	-
	chr4:16000112-16000112	PROM1 c.1579-1G>C	het	novel	-
**12010986**	chr14:21794230-21794231	RPGRIP1 c.2608_2609insA	hom	novel	-

Supp. Table S5. Genetic variants reported to referring clinicians, including causative, likely causative, and variants of unknown clinical significance.

**MCGM ID**	**Genomic loci** ***hg19***	**Variant details** ***specified transcripts, Table S1***	**Zygosity**	**Supporting evidence from literature**	
				**Disease segregation**	**Functional**
**13008348**	chr2:182468670-182468670	CERKL c.375C>G p.(Cys125Trp)	hom	[3, 4]	[5]
**11010719**	chr2:182423344-182423344	CERKL c.847C>T p.(Arg283Ter)	hom	[3]	
**Patients referred with FEVR**					
**13010229**[+]	chr11:68115344-68115344	LRP5 c.121C>T p.(Arg41Trp)	het	novel	-
	chr11:68153802-68153802	LRP5 c.1034T>A p.(Leu345Gln)	het	novel	-
**12006042**[+]	chr11:86662513-86662516	FZD4 c.1282_1285delGACA	het	[59]	-
	chr11:86662837-86662837	FZD4 c.961G>A p.(Val321Ile)	het	novel	-
**12000347**	chr11:86663485-86663485	FZD4 c.313A>G p.(Met105Val)	het	[60, 61]	-
**Patients referred with JOUBERT**					
**13009772**[+]	chr12:88471040-88471040	CEP290 c.5668G>T p.(Gly1890Ter)	hom	[62]	-
**Patients referred with EORD/LCA**					
**13015227**	chr1:68905008-68905008	RPE65 c.726-2A>C	hom	novel	-
**83830**[+]	chr17:7919559-7919559	GUCY2D c.3175A>T p.(Arg1059Ter)	het	novel	-
	chr17:7909706-7909706	GUCY2D c.1052A>G p.(Tyr351Cys)	het	[63, 64]	-
**12012655**[+]	chr14:68192807-68192807	RDH12 c.383T>G p.(Val128Gly)	het	novel	-
	chr14:68200524-68200524	RDH12 c.910T>C p.(Trp304Arg)	het	novel	-
**13009681**[+]	chr1:197404030-197404030	CRB1 c.3037C>T p.(Gln1013Ter)	het	[33]	
	chr1:197396856-197396856	CRB1 c.2401A>T p.(Lys801Ter)	het	[33, 65]	-
**13017541**	chr17:7918821-7918821	GUCY2D c.2944+1delG	hom	[21-23]	-

Supp. Table S5. Genetic variants reported to referring clinicians, including causative, likely causative, and variants of unknown clinical significance.

MCGM ID	Genomic loci *hg19*	Variant details *specified transcripts, Table S1*	Zygosity	Supporting evidence from literature	
				Disease segregation	Functional
**13001467**	chr12:88478404-88478406	CEP290 c.4661_4663delAAG p.(Glu1554del)	het	[66, 67]	-
	chr12:88532921-88532921	CEP290 c.297+1G>T	het	novel	-
**12009612**	chr14:21816333-21816333	RPGRIP1 c.3620T>G p.(Leu120Ter)	hom	[68]	-
**11011508**	chrX:46713068-46713076	RP2 c.260_268delCTAACTGCA p.(Thr87_Cys89del)	hemi	novel	-
**13010893**	chr12:88465563-88465563	CEP290 c.5850delT	het	[67, 69]	-
	chr12:88477713-88477713	CEP290 c.4723A>T p.(Lys1575Ter)	het	[67, 69]	-
**13015209**	chr17:7918821-7918821	GUCY2D c.2944+1delG	hom	[21-23]	-
**13003353**+	chr12:88494960-88494960	CEP290 c.2991+1655A>G	het	[70]	-
	chr12:88510853-88510853	CEP290 c.1781T>A p.(Leu594Ter)	het	novel	-
**13009682**	chr1:197404007-197404007	CRB1 c.3014A>T p.(Asp1005Val)	het	[35]	-
	chr1:197396856-197396856	CRB1 c.2401A>T p.(Lys801Ter)	het	[33, 65]	-
**11013818**+	chr1:197403836-197403836	CRB1 c.2843G>A p.(Cys948Tyr)	hom	[33-37]	mutation hotspot [34]
**12003872**	chr6:80223010-80223016	LCA5 c.633_639delAGCTAGA	hom	novel	-
**13001571**+	chr1:197403836-197403836	CRB1 c.2843G>A p.(Cys948Tyr)	het	[33-37]	mutation hotspot [34]
	chr1:197298065-197298065	CRB1 c.584G>T p.(Cys195Phe)	het	[34]	-
**13013813**	chr14:21793021-21793021	RPGRIP1 c.2007delT	hom	novel	-
**12012994**	chr17:6337250-6337250	AIPL1 c.265T>C p.(Cys89Arg)	hom	[71, 72]	[71]
**12013890**	chr1:68906634-68906634	RPE65 c.545A>G p.(His182Arg)	hom	[73, 74]	p.His182Tyr, p.His182Asn [74]
**13018022**	chr14:21792906-21792906	RPGRIP1 c.1892A>G p.(His631Arg)	hom	p.(His631Pro) [75]	[76]

Supp. Table S5. Genetic variants reported to referring clinicians, including causative, likely causative, and variants of unknown clinical significance.

**MCGM ID**	**Genomic loci** ***hg19***	**Variant details** ***specified transcripts, Table S1***	**Zygosity**	**Supporting evidence from literature**	
				**Disease segregation**	**Functional**
**13005787**	chr17:7918737-7918737	GUCY2D c.2861T>C p.(Leu954Pro)	hom	[77, 78]	[22]
**12015787**+	chr14:21780621-21780621	RPGRIP1 c.1107delA	hom	[79]	-
**12001399**	chr1:197404067-197404067	CRB1 c.3074G>A p.(Ser1025Asn)	hom	[33] p.(Ser1025Ile) [63]	-
**13002979**+	chr4:16008260-16008261	PROM1 c.1354dupT	het	[47]	-
	chr4:16025914-16025914	PROM1 c.694+4A>T	het	novel	-
**13003494**	chr1:68910508-68910508	RPE65 c.304G>T p.(Glu102Ter)	hom	[80-82]	-
**12013231**+	chr19:48342890-48342890	CRX c.566delC	het	other final exon frameshift mutations [83, 84]	-
**12012257**	chr14:88904207-88904218	SPATA7 c.1241_1252delTCCTGAAAGTAG p.(Val414_Val417del)	het	novel	-
	chr14:88897545-88897545	SPATA7 c.1058dupC	het	novel	-
**13005796**	chr11:76885842-76885842	MYO7A c.1976C>A p.(Ser659Ter)	het	novel	-
	chr11:76892594-76892594	MYO7A c.2863G>A p.(Gly955Ser)	het	[85, 86]	-
**12004275**	chr4:155665951-155665951	LRAT c.473G>A p.(Trp158Ter)	hom	novel	-
**12013710**	chr12:88494960-88494960	CEP290 c.2991+1655A>G	het	[70]	-
	chr12:88474003-88474003	CEP290 c.5182G>T p.(Glu1728Ter)	het	[62, 87]	-
**11010486**	chr1:197403836-197403836	CRB1 c.2843G>A p.(Cys948Tyr)	het	[33-37]	mutation hotspot [34]
	chr1:197398590-197398590	CRB1 c.2688T>A p.(Cys896Ter)	het	[33, 63, 88]	-
**13007873**	chr1:197403862-197403862	CRB1 c.2869C>T p.(Gln957Ter)	het	novel	-
	chr1:197403836-197403836	CRB1 c.2843G>A p.(Cys948Tyr)	het	[33-37]	mutation hotspot [34]
**12006834**+	chr12:88494960-88494960	CEP290 c.2991+1655A>G	het	[70]	-

Supp. Table S5. Genetic variants reported to referring clinicians, including causative, likely causative, and variants of unknown clinical significance.

**MCGM ID**	**Genomic loci** *hg19*	**Variant details** *specified transcripts, Table S1*	**Zygosity**	**Supporting evidence from literature**	
				**Disease segregation**	**Functional**
	chr12:88477635-88477635	CEP290 c.4801C>T p.(Gln1601Ter)	het	novel	-
**12009433**+	chr12:88494960-88494960	CEP290 c.2991+1655A>G	het	[70]	-
	chr12:88508195-88508196	CEP290 c.2052+1_2052+2delGT	het	novel	-
**13005926**	chr12:88519134-88519134	CEP290 c.1078C>T p.(Arg360Ter)	het	[66]	-
	chr12:88477713-88477713	CEP290 c.4723A>T p.(Lys1575Ter)	het	[67, 69]	-
**12008985**	chr1:68912507-68912507	RPE65 c.131G>A p.(Arg44Gln)	hom	[89]	[90, 91]
**13002684**	chr17:7918821-7918821	GUCY2D c.2944+1delG	hom	[21-23]	-
**13005789**	chr14:68193831-68193831	RDH12 c.582C>G p.(Tyr194Ter)	het	[92, 93]	-
	chr14:68196099-68196099	RDH12 c.848+2T>C	het	novel	-
**12014872**	chr1:197397003-197397003	CRB1 c.2548G>A p.(Gly850Ser)	het	[33, 34]	mutation hotspot [34]
	chr1:197403836-197403836	CRB1 c.2843G>A p.(Cys948Tyr)	het	[33-37]	mutation hotspot [34]
**13010086**	chr14:21780621-21780621	RPGRIP1 c.1107delA	hom	[79]	-
**12004181**	chr12:88487674-88487675	CEP290 c.3181_3182delAT	het	novel	-
	chr12:88524986-88524986	CEP290 c.451C>T p.(Arg151Ter)	het	[94]	-
**13015422**	chr17:6329101-6329101	AIPL1 c.834G>A p.(Trp278Ter)	hom	[95]	[96]
**13005791**	chr14:68193773-68193773	RDH12 c.524C>T p.(Ser175Leu)	het	[69]	p.(Ser175Pro) [97]
	chr14:68193897-68193927	RDH12 c.648_658+20delGAGGCTCCAAGGTAAGTCTGGAGAAAGAGGA	het	novel	-
**13002515**	chr14:21786006-21786006	RPGRIP1 c.1303A>T p.(Lys435Ter)	hom	novel	-
**12007217**+	chr12:88494960-88494960	CEP290 c.2991+1655A>G	het	[70]	-
	chr12:88476854-88476854	CEP290 c.4966G>T p.(Glu1656Ter)	het	[70]	-
**13012618**	chr1:197396856-197396856	CRB1 c.2401A>T p.(Lys801Ter)	het	[33, 65]	-
	chr1:197390570-197390571	CRB1 c.1612_1613insCTTA	het	novel	-

Supp. Table S5. Genetic variants reported to referring clinicians, including causative, likely causative, and variants of unknown clinical significance.

MCGM ID	Genomic loci *hg19*	Variant details *specified transcripts, Table S1*	Zygosity	Supporting evidence from literature	
				Disease segregation	Functional
**13002689**	chr14:21788314-21788314<br>chr14:21793489-21793489	RPGRIP1 c.1445T>A p.(Leu482Ter)<br>RPGRIP1 c.2314C>T p.(Gln772Ter)	het<br>het	novel<br>novel	-<br>-
**12007024**+	chr1:197403835-197403835<br>chr1:197396856-197396856	CRB1 c.2843-1G>A<br>CRB1 c.2401A>T p.(Lys801Ter)	het<br>het	novel<br>[33, 65]	-<br>-
**13002649**	chr17:7918821-7918821	GUCY2D c.2944+1delG	hom	[21-23]	-
**13005797**	chr17:7906745-7906745<br>chr17:7918195-7918195	GUCY2D c.380C>T p.(Pro127Arg)<br>GUCY2D c.2595delG	het<br>het	novel<br>[98]	-
**Patients referred with OTHER**					
**12010090**	chr2:170336065-170336065	BBS5 c.2T>A p.(Met1?)	hom	[99]	-
**13010232**+	chr12:56115698-56115698	RDH5 c.536A>G p.(Lys179Arg)	hom	novel	mutation hotspot [100]
**Patients referred with RP/RCD**					
**13003141**	chr2:96958829-96958829	SNRNP200 c.2041C>T p.(Arg681Cys)	het	[101]	-
**12013732**+	chr8:55538040-55538043	RP1 c.1598_1601delGAAA	het	novel	-
**12003878**	chr17:1564976-1564976	PRPF8 c.4131C>T p.(Ser1377Ser)	het	novel	-
**13001452**	chr2:112761560-112761560	MERTK c.1866G>C p.(Lys622Asn)	hom	novel	-
**12011770**	chr4:16000058-16000058<br>chr4:15995680-15995680	PROM1 c.1632G>T p.(Gly544Gly)<br>PROM1 c.1697delA	het<br>het	novel<br>novel	-<br>-
**13015513**	chr8:55534803-55534803	RP1 c.742C>T p.(Gln248Ter)	het	novel	location p.500-p.1053 [102]

Supp. Table S5. Genetic variants reported to referring clinicians, including causative, likely causative, and variants of unknown clinical significance.

MCGM ID	Genomic loci *hg19*	Variant details *specified transcripts, Table S1*	Zygosity	Supporting evidence from literature	
				Disease segregation	Functional
	chr8:55534065-55534065	RP1 c.539T>G p.(Phe180Cys)	het	novel	-
**12015109**	chr2:112779023-112779023	MERTK c.2214delT	hom	[103]	-
**13002962**+	chr2:182468729-182468729	CERKL c.316C>T p.(Arg106Cys)	het	p.(Arg106Ser) [104]	-
	chr2:182468670-182468670	CERKL c.375C>G p.(Cys125Trp)	het	[3, 4]	[5]
**13015666**+	chr1:215847812-215847812	USH2A c.13441A>G p.(Arg4481Gly)	het	novel	-
	chr1:216062399-216062399	USH2A c.7595-3C>G	het	[105-107]	[107]
**11000824**+	chr1:197396763-197396763	CRB1 c.2308G>A p.(Gly770Ser)	het	novel	-
	chr1:197396856-197396856	CRB1 c.2401A>T p.(Lys801Ter)	het	[33, 65]	-
**11005352**	chr19:54631496-54631496	PRPF31 c.994C>T p.(Gln332Ter)	het	novel	-
**13003075**	chr3:150645894-150645894	CLRN1 c.567T>G p.(Tyr189Ter)	het	[108, 109]	-
	chr3:150659443-150659443	CLRN1 c.359T>A p.(Met120Lys)	het	[109]	[110]
**12014873**	chr3:100962762-100962763	IMPG2 c.2412_2413delTG	het	novel	-
	chr3:100947720-100947720	IMPG2 c.3634G>T p.(Glu1212Ter)	het	novel	-
**13012950**+	chr15:72103895-72103895	NR2E3 c.191G>T p.(Cys64Phe)	het	novel	-
	chr15:72103821-72103821	NR2E3 c.119-2A>C	het	[111-113]	[114]
**12000462**	chr1:216424273-216424273	USH2A c.2139C>T p.(Gly713Gly)	het	novel	-
	chr1:216462734-216462734	USH2A c.1859G>T p.(Cys620Phe)	het	[115]	-
**13010985**+	chrX:46737028-46737028	RP2 c.969+3A>G	hemi	[116, 117]	[117]
**12002962**	chr10:55582113-55582122	PCDH15 c.5385_5394delTCCTCTTCCT	het	novel	-
	chr10:55581921-55581921	PCDH15 c.5586C>A p.(Ala1862Ala)	het	novel	-
**13004711**	chr8:55538471-55538471	RP1 c.2029C>T p.(Arg677Ter)	het	[118, 119]	-
**13012171**	chrX:85155656-85155656	CHM c.1408C>T p.(Gln470Ter)	hemi	[120]	-
**12012050**	chr16:57998062-57998062	CNGB1 c.262C>T p.(Gln88Ter)	het	novel	-
	chr16:57938727-57938728	CNGB1 c.2544dupG	het	novel	-

Supp. Table S5. Genetic variants reported to referring clinicians, including causative, likely causative, and variants of unknown clinical significance.

MCGM ID	Genomic loci *hg19*	Variant details *specified transcripts, Table S1*	Zygosity	Supporting evidence from literature	
				Disease segregation	Functional
**13010307**	chr2:62066572-62066572	FAM161A c.1567C>T p.(Arg523Ter)	het	[121]	-
	chr2:62066830-62066830	FAM161A c.1309A>T p.(Arg437Ter)	het	[122]	-
**12010984**+	chr2:96953706-96953706	SNRNP200 c.3260C>T p.(Ser1087Leu)	het	[101, 123]	[123, 124]
**13006766**	chr19:54626942-54626942	PRPF31 c.527+3A>G	het	[125, 126]	-
**13017314**	chrX:38158210-38158211	RPGR c.1243_1244delAG	hemi	[127]	-
**13003723**	chr1:216221921-216221921	USH2A c.6118T>G p.(Cys2040Gly)	het	novel	-
	chr1:216373373-216373373	USH2A c.3407G>A p.(Ser1136Asn)	het	[115]	-
**10003544**	chr16:57935275-57935275	CNGB1 c.2957A>T p.(Asn986Ile)	hom	[39]	-
**13007749**	chr1:216420460-216420460	USH2A c.2276G>T p.(Cys759Phe)	het	[128-132]	-
	chr1:216420437-216420437	USH2A c.2299delG	het	[27, 115, 133-136]	-
**12007876**	chr3:129251131-129251131	RHO c.568G>A p.(Asp190Asn)	het	[137]	[138]
**13013774**	chr1:197396745-197396745	CRB1 c.2290C>T p.(Arg764Cys)	het	[33, 35, 36]	-
	chr1:197298065-197298065	CRB1 c.584G>T p.(Cys195Phe)	het	[34]	-
**12002958**	chr6:64430796-64430796	EYS c.9131G>T p.(Trp3044Leu)	het	[139]	-
	chr6:65016917-65016917	EYS c.6137G>A p.(Trp2046Ter)	het	novel	-
**12013881**	chr14:68193850-68193850	RDH12 c.601T>C p.(Cys201Arg)	hom	[140]	[140]
**13006275**	chr9:32541984-32541984	TOPORS c.2539C>T p.(Arg847Ter)	het	[27]	-
**12002573**	chr6:42141500-42141500	GUCA1A c.149C>T p.(Pro50Leu)	het	[141]	[142]
**12016026**+	chr1:197446947-197446947	CRB1 c.4159G>T p.(Glu1387Ter)	het	novel	-
	chr1:197398590-197398590	CRB1 c.2688T>A p.(Cys896Ter)	het	[33, 63, 88]	-
**13002431**+	chr19:54625894-54625894	PRPF31 c.341T>A p.(Ile114Asn)	het	novel	-
**12007088**	chr4:658734-658734	PDE6B c.2193+1G>A	het	[143]	-
	chr4:619706-619706	PDE6B c.291C>A p.(Tyr97Ter)	het	novel	-

Supp. Table S5. Genetic variants reported to referring clinicians, including causative, likely causative, and variants of unknown clinical significance.

MCGM ID	Genomic loci *hg19*	Variant details *specified transcripts, Table S1*	Zygosity	Supporting evidence from literature	
				Disease segregation	Functional
**12005728**	chr19:54625953-54625953	PRPF31 c.400delG	het	novel	-
**13010253**	chr14:24550532-24550573	NRL c.586_627dupGCCCAGCTGGACGCGCTGCGGGCC GAGGTGGCCCGCCTGGCC p.(Ala196_Ala209dup)	het	novel	-
**13011161**	chrX:38164014-38164014	RPGR c.808C>T p.(Gln270Ter)	hemi	novel	-
**13012574**	chr3:129252554-129252554	RHO c.1040C>T p.(Pro347Leu)	het	[144, 145]	[146]
**13004232**[+]	chr4:16014897-16014897<br>chr4:16000058-16000058	PROM1 c.1141+1G>A<br>PROM1 c.1632G>T p.(Gly544Gly)	het<br>het	novel<br>novel	-<br>-
**13015639**	chr1:94528164-94528164<br>chr1:94517254-94517254	ABCA4 c.1906C>T p.(Gln636Ter)<br>ABCA4 c.2588G>C p.(Gly863Ala)	het<br>het	[147, 148]<br>[11, 149]	-<br>-
**12016035**	chr1:94506945-94506945	ABCA4 c.3342delC	hom	[49]	
**12002868**[+]	chr8:55539055-55539055	RP1 c.2613dupA	het	[150]	
**12014047**[+]	chr1:197396764-197396764<br>chr1:197404010-197404010	CRB1 c.2309G>A p.(Gly770Asp)<br>CRB1 c.3017C>T p.(Ser1006Phe)	het<br>het	novel<br>novel	-<br>-
**11006153**	chr2:62066675-62066675	FAM161A c.1464G>A p.(Trp488Ter)	hom	novel	-
**13011266**	chrX:46713315-46713315	RP2 c.507delT	hemi	novel	-
**13004716**	chr6:65612025-65612026	EYS c.2826_2827delAT	hom	novel	-
**12010854**	chrX:85302485-85302485	CHM c.49+3A>G	hemi	c.49+5G>T [151]	c.49+5G>T [151]
**13000107**	chr2:112725802-112725804<br>chr2:112777090-112777090	MERTK c.933_935delinsTT<br>MERTK c.2180G>A p.(Arg727Gln)	het<br>het	novel<br>novel	-<br>-
**13002976**[+]	chr10:85956379-85956379	CDHR1 c.270dupC	hom	novel	-
**13004227**	chr8:55538474-55538474	RP1 c.2032C>T p.(Gln678Ter)	het	novel	-

Supp. Table S5. Genetic variants reported to referring clinicians, including causative, likely causative, and variants of unknown clinical significance.

MCGM ID	Genomic loci *hg19*	Variant details *specified transcripts, Table S1*	Zygosity	Supporting evidence from literature	
				Disease segregation	Functional
**13000106**	chr4:47938971-47938971	CNGA1 c.1747C>T p.(Arg583Ter)	hom	novel	-
**13008977**	chr3:129252554-129252554	RHO c.1040C>T p.(Pro347Leu)	het	[144, 145]	[146]
**10003578**	chr1:215955512-215955512<br>chr1:216424331-216424331	USH2A c.10612C>T p.(Arg3538Ter)<br>USH2A c.2081G>C p.(Cys694Ser)	het<br>het	novel<br>p.Cys694Tyr [38]	-<br>-
**12015280**	chr1:216420460-216420460<br>chr1:215848679-215848679	USH2A c.2276G>T p.(Cys759Phe)<br>USH2A c.12574C>T p.(Arg4192Cys)	het<br>het	[128-132]<br>p.Arg4192His [152, 153]	-<br>-
**13010104**	chrX:46696638-46696638	RP2 c.102+1G>A	hemi	[154]	-
**12002571**	chr17:1565308-1565312	PRPF8 c.3910_3914delAACTC	het	novel	-
**13004912**	chr1:216420460-216420460<br>chr1:216420437-216420437	USH2A c.2276G>T p.(Cys759Phe)<br>USH2A c.2299delG	het<br>het	[128-132]<br>[27, 115, 133-136]	-<br>-
**13007240**+	chr6:65655758-65655758	EYS c.2309A>C p.(Gln770Pro)	hom	[155]	-
**13010305**	chr19:54629963-54629963	PRPF31 c.916G>A p.(Asp306Asn)	het	novel	location [156]
**13002044**+	chr6:66112400-66112400	EYS c.1155T>A p.(Cys385Ter)	hom	novel	-
**12013544**+	chr2:182468670-182468670<br>chr2:182521541-182521541	CERKL c.375C>G p.(Cys125Trp)<br>CERKL c.193G>T p.(Glu65Ter)	het<br>het	[3, 4]<br>novel	[5]<br>-
**12002963**	chr6:66204814-66204814	EYS c.490C>T p.(Arg164Ter)	hom	novel	-
**12011781**	chr1:216420460-216420460<br>chr1:215914880-215914880	USH2A c.2276G>T p.(Cys759Phe)<br>USH2A c.11549-1G>A	het<br>het	[128-132]<br>novel	-<br>-
**13016075**	chrX:46713161-46713161	RP2 c.353G>A p.(Arg118His)	hemi	[157]	[158]
**12011157**	chr17:1554143-1554143	PRPF8 c.6961C>T p.(Gln2321Ter)	het	novel	-
**13018012**+	chr1:216420460-216420460	USH2A c.2276G>T p.(Cys759Phe)	het	[128-132]	-

Supp. Table S5. Genetic variants reported to referring clinicians, including causative, likely causative, and variants of unknown clinical significance.

MCGM ID	Genomic loci *hg19*	Variant details *specified transcripts, Table S1*	Zygosity	Supporting evidence from literature	
				Disease segregation	Functional
	chr1:216498841-216498841	USH2A c.949C>A p.(Arg317Arg)	het	[159, 160]	[161]
**13005635**+	chrX:38163914-38163914	RPGR c.908G>A p.(Gly303Glu)	hemi	novel	-
**12015561**+	chr3:129251104-129251104	RHO c.541G>A p.(Glu181Lys)	het	[162-164]	[165]
**12002960**	chr2:62066830-62066830	FAM161A c.1309A>T p.(Arg437Ter)	hom	[122]	-
**12002727**+	chr22:33255338-33255338	TIMP3 c.610A>T p.(Ser204Cys)	het	[166]	-
**12012775**	chr2:96959219-96959219	SNRNP200 c.1871G>A p.(Arg624Lys)	het	novel	-
**12005771**+	chr1:197313565-197313565	CRB1 c.807dupA	het	novel	-
	chr1:197398590-197398590	CRB1 c.2688T>A p.(Cys896Ter)	het	[33, 63, 88]	-
**13012959**	chr16:57935339-57935339	CNGB1 c.2893G>A p.(Gly965Ser)	het	novel	-
	chr16:57965773-57965773	CNGB1 c.1382C>T p.(Thr461Met)	het	novel	-
**13007688**	chr2:96955598-96955598	SNRNP200 c.2879C>T p.(Ala960Val)	het	novel	-
**13007207**	chr1:68912520-68912520	RPE65 c.118G>A p.(Gly40Ser)	hom	[91, 167]	-
**13004358**	chr5:89975426-89975429	GPR98 c.5504_5507delTTCC	hom	novel	-
**13015421**	chr2:98996670-98996670	CNGA3 c.248G>A p.(Trp83Ter)	het	novel	-
	chr2:99013564-99013564	CNGA3 c.1931T>C p.(Phe644Ser)	het	novel	-
**075849**+	chr4:16002140-16002140	PROM1 c.1557C>A p.(Tyr519Ter)	het	[168]	-
	chr4:16008260-16008261	PROM1 c.1354dupT	het	[47]	-
**13008349**	chr17:1554982-1554982	PRPF8 c.6470T>A p.(Val2157Glu)	het	novel	-
**13018110**	chr3:150690352-150690352	CLRN1 c.144T>G p.(Asn48Lys)	hom	[169, 170]	[110]
**13009346**	chr4:47942803-47942803	CNGA1 c.848A>T p.(Asp283Val)	het	novel	-
	chr4:47938964-47938964	CNGA1 c.1754T>G p.(Met585Arg)	het	novel	-
**11006503**	chr17:7918017-7918018	GUCY2D c.2511_2512delinsCA p.(Glu837_Arg838delinsAspSer)	het	[8, 171, 172]	-

Supp. Table S5. Genetic variants reported to referring clinicians, including causative, likely causative, and variants of unknown clinical significance.

MCGM ID	Genomic loci *hg19*	Variant details *specified transcripts, Table S1*	Zygosity	Supporting evidence from literature	
				Disease segregation	Functional
**13004487**+	chr6:66112400-66112400	EYS c.1155T>A p.(Cys385Ter)	hom	novel	-
**12002965**+	chr12:88479860-88479860	CEP290 c.4393C>T p.(Arg1465Ter)	het	[173]	-
	chr12:88534765-88534765	CEP290 c.148C>T p.(His50Tyr)	het	novel	-
**13002499**	chr19:54631469-54631469	PRPF31 c.967G>T p.(Glu323Ter)	het	novel	-
**12008423**+	chr1:216369924-216369924	USH2A c.4222C>T p.(Gln1408Ter)	hom	[134]	-
**13012899**	chrX:38176607-38176607	RPGR c.581G>A p.(Trp194Ter)	hemi	[174, 175]	-
**12011769**	chr2:182413512-182413513	CERKL c.1045_1046delAT	hom	novel	-
**13005790**	chr2:112779111-112779111	MERTK c.2302G>A p.(Ala768Thr)	hom	novel	-
**13001457**	chr8:55538727-55538731	RP1 c.2285_2289delTAAAT	het	[118]	-
**13016341**	chr3:129249866-129249866	RHO c.509C>G p.(Pro170Arg)	het	[176, 177]	-
**13017339**	chr1:197396856-197396856	CRB1 c.2401A>T p.(Lys801Ter)	hom	[33, 65]	-
**13018482**	chr4:16025917-16025917	PROM1 c.694+1G>A	het	novel	-
	chr4:16000112-16000112	PROM1 c.1579-1G>C	het	novel	-
**13009597**+	chr2:96958828-96958828	SNRNP200 c.2042G>A p.(Arg681Cys)	het	[101]	-
**13003909**+	chr1:197398735-197398735	CRB1 c.2833G>A p.(Gly945Arg)	het	novel	-
	chr1:197403836-197403836	CRB1 c.2843G>A p.(Cys948Tyr)	het	[33-37]	mutation hotspot [34]
**13013461**	chr1:94463488-94463488	ABCA4 c.6658C>T p.(Gln2220Ter)	hom	[52-54]	-
**13018544**	chrX:38150211-38150211	RPGR c.1572+1delG	hemi	novel	-
**13003354**	chr1:197396820-197396822	CRB1 c.2365_2367delAAT p.(Asn789del)	het	[178]	-
	chr1:197403836-197403836	CRB1 c.2843G>A p.(Cys948Tyr)	het	[33-37]	mutation hotspot [34]
**12002961**	chr3:101039148-101039149	IMPG2 c.68dupA	hom	novel	-

Supp. Table S5. Genetic variants reported to referring clinicians, including causative, likely causative, and variants of unknown clinical significance.

**MCGM ID**	**Genomic loci** ***hg19***	**Variant details** ***specified transcripts, Table S1***	**Zygosity**	**Supporting evidence from literature**	
				**Disease segregation**	**Functional**
**13013069**	chr2:182468670-182468670	CERKL c.375C>G p.(Cys125Trp)	hom	[3, 4]	[5]
**13008037+**	chr1:216221935-216221935<br>chr1:216420437-216420437	USH2A c.6104G>A p.(Cys2035Tyr)<br>USH2A c.2299delG	het<br>het	novel<br>[27, 115, 133-136]	-<br>-
**12011625**	chr8:55537904-55537904	RP1 c.1462delG	hom	novel	-
**12010657+**	chr14:57268813-57268813	OTX2 c.534C>A p.(Cys178Ter)	het	[179]	-
**13000499+**	chr2:182468670-182468670<br>chr2:182521541-182521541	CERKL c.375C>G p.(Cys125Trp)<br>CERKL c.193G>T p.(Glu65Ter)	het<br>het	[3, 4]<br>novel	[5]<br>-
**12008984+**	chr11:61725751-61725753	BEST1 c.848_850delTTC p.(Phe283Ter)	het	[180]	-
**13013377**	chr6:66112400-66112400	EYS c.1155T>A p.(Cys385Ter)	hom	novel	-
**12002569**	chr11:119210284-119210284	C1QTNF5 c.489C>A p.(Ser163Arg)	het	c.489C>G [181]	-
**12002956**	chr14:88883069-88883069	SPATA7 c.253C>T p.(Arg85Ter)	hom	[182]	-
**13012788**	chr1:216420460-216420460<br>chr1:216380743-216380744	USH2A c.2276G>T p.(Cys759Phe)<br>USH2A c.3187_3188delCA	het<br>het	[128-132]<br>[134]	-<br>-
**11011655+**	chr19:54631562-54631562	PRPF31 c.1060C>T p.(Arg354Ter)	het	novel	-
**13006601**	chr3:129251479-129251479	RHO c.800C>T p.(Pro267Leu)	het	[177, 183]	-
**12010631+**	chr8:55538471-55538471	RP1 c.2029C>T p.(Arg677Ter)	het	[118, 119]	-
**12015182**	chr1:215963510-215963510	USH2A c.10073G>A p.(Cys3358Tyr)	hom	[115, 132, 152, 153, 155]	-
**13004890+**	chr3:100963495-100963495	IMPG2 c.1680T>A p.(Tyr560Ter)	hom	[121]	-
**13002513**	chr5:149323870-149323870<br>chr5:149263008-149263008	PDE6A c.367G>T p.(Asp123Tyr)<br>PDE6A c.2119C>T p.(Arg707Trp)	het<br>het	novel<br>novel	-<br>-
**13000500+**	chr16:57935275-57935275	CNGB1 c.2957A>T p.(Asn986Ile)	het	[39]	-

Supp. Table S5. Genetic variants reported to referring clinicians, including causative, likely causative, and variants of unknown clinical significance.

MCGM ID	Genomic loci *hg19*	Variant details *specified transcripts, Table S1*	Zygosity	Supporting evidence from literature	
				Disease segregation	Functional
	chr16:57937839-57937839	CNGB1 c.2681G>A p.(Arg894His)	het	novel	-
	chr16:57951242-57951242	CNGB1 c.2096A>G p.(Asp699Gly)	het	novel	-
**13007209**	chr8:55539191-55539191	RP1 c.2749C>T p.(Gln917Ter)	het	novel	-
**12002277**	chr1:197297979-197297990	CRB1 c.498_506delAATTGATGGTTA p.(Ile167_Gly169del)	het	[35]	
	chr1:197396584-197396584	CRB1 c.2129A>T p.(Glu710Val)	het	[33, 38, 39]	-
**13000103**	chrX:38182652-38182652	RPGR c.154G>A p.(Gly52Arg)	hemi	[184, 185]	[186]
**Patients referred with MD/STGD**					
**12008814**	chr1:94514477-94514477	ABCA4 c.2690C>T p.(Thr897Ile)	het	[44, 187-190]	-
	chr1:94544977-94544977	ABCA4 c.1140T>A p.(Asn380Lys)	het	[44, 187, 189]	-
**13012938**[+]	chr17:6329101-6329101	AIPL1 c.834G>A p.(Trp278Ter)	het	[95]	[96]
**13017782**[+]	chr8:55533647-55533647	RP1 c.121T>C p.(Tyr41His)	het	novel	-
	chr8:55534041-55534041	RP1 c.515T>G p.(Leu172Arg)	het	[155]	-
**13002608**	chr1:94528164-94528164	ABCA4 c.1906C>T p.(Gln636Ter)	het	[147, 148]	-
	chr1:94496666-94496666	ABCA4 c.4139C>T p.(Pro1380Leu)	het	[12, 45, 48, 49, 191]	[50]
**13014675**	chr1:94496010-94496010	ABCA4 c.4326C>A p.(Asn1442Lys)	het	[141, 147, 149]	-
	chr1:94471065-94471065	ABCA4 c.6079C>T p.(Leu2027Phe)	het	[12]	[13]
**13016023**	chr11:61726979-61726979	BEST1 c.877C>A p.(Gln293Lys)	het	[192]	p.(Gln293His) [193]
**13008350**	chr2:182521541-182521541	CERKL c.193G>T p.(Glu65Ter)	hom	novel	-
**13012365**	chr1:94473807-94473807	ABCA4 c.5882G>A p.(Gly1961Glu)	het	[11, 49, 194, 195]	[50]
	chr1:94528197-94528197	ABCA4 c.1873C>T p.(Gln625Ter)	het	novel	-

Supp. Table S5. Genetic variants reported to referring clinicians, including causative, likely causative, and variants of unknown clinical significance.

MCGM ID	Genomic loci *hg19*	Variant details *specified transcripts, Table S1*	Zygosity	Supporting evidence from literature	
				Disease segregation	Functional
**13003074**	chr2:182468670-182468670	CERKL c.375C>G p.(Cys125Trp)	hom	[3, 4]	[5]
**13012308**	chr19:48342929-48342929	CRX c.605delG	het	novel	-
**13013879**	chr1:94508386-94508386	ABCA4 c.3259G>A p.(Glu1087Lys)	hom	[10]	-
**13001831**+	chr1:94490567-94490567	ABCA4 c.4577C>T p.(Thr1526Met)	het	[45, 48]	-
	chr1:94486794-94486794	ABCA4 c.5018+2T>C	het	[45, 188]	-
**12005906**	chr1:94473807-94473807	ABCA4 c.5882G>A p.(Gly1961Glu)	het	[11, 49, 194, 195]	[50]
	chr1:94496666-94496666	ABCA4 c.4139C>T p.(Pro1380Leu)	het	[12, 45, 48, 49, 191]	[50]
**13009479**	chr6:42672272-42672272	PRPH2 c.659G>A p.(Asn220Gln)	het	p.(Arg220Trp) [196]	-
**13012619**	chr1:94471037-94471037	ABCA4 c.6107A>G p.(Tyr2036Cys)	het	novel	-
	chr1:94495071-94495071	ABCA4 c.4469G>A p.(Cys1490Tyr)	het	[46, 197, 198]	[197]
**13009064**+	chr19:48337801-48337801	CRX c.100+1G>C	het	novel	-
**13017396**	chr11:61723244-61723244	BEST1 c.302C>T p.(Pro101Leu)	het	[199] p.(Pro101Thr) [180, 200]	-
	chr11:61723364-61723364	BEST1 c.422G>A p.(Arg141His)	het	[180, 201-205]	[206]
**12013892**	chr9:2729470-2729470	KCNV2 c.1381G>A p.(Gly461Arg)	hom	[207-209]	-
**12001090**	chr1:94528819-94528819	ABCA4 c.1609C>T p.(Arg537Cys)	hom	[49]	-
**13011741**	chr2:182468670-182468670	CERKL c.375C>G p.(Cys125Trp)	hom	[3, 4]	[5]
**12004145**	chr1:94508969-94508969	ABCA4 c.3113C>T p.(Ala1038Val)	het	[11, 210]	[50]
	chr1:94577135-94577135	ABCA4 c.161G>A p.(Cys54Tyr)	het	[45, 48, 195]	-
**13015690**	chr1:94473807-94473807	ABCA4 c.5882G>A p.(Gly1961Glu)	het	[11, 49, 194, 195]	[50]
	chr1:94520688-94520688	ABCA4 c.2566T>A p.(Tyr856Asn)	het	novel	-

Supp. Table S5. Genetic variants reported to referring clinicians, including causative, likely causative, and variants of unknown clinical significance.

MCGM ID	Genomic loci *hg19*	Variant details *specified transcripts, Table S1*	Zygosity	Supporting evidence from literature	
				Disease segregation	Functional
**13006449**	chr2:182468670-182468670	CERKL c.375C>G p.(Cys125Trp)	het	[3, 4]	[5]
	chr2:182521541-182521541	CERKL c.193G>T p.(Glu65Ter)	het	novel	-
**Patients referred with USHER**					
**12015998**	chr11:76924019-76924019	MYO7A c.6377delC	hom	[115]	-
**12010758+**	chr1:216420460-216420460	USH2A c.2276G>T p.(Cys759Phe)	het	[128-132]	-
	chr1:215847475-215847475	USH2A c.13778C>T p.(Ser4593Leu)	het	novel	-
**13009036**	chr1:216373453-216373453	USH2A c.3327C>A p.(Tyr1109Ter)	het	novel	-
	chr1:216420437-216420437	USH2A c.2299delG	het	[27, 115, 133-136]	-
**12007911**	chr1:216270451-216270451	USH2A c.4732C>T p.(Arg1578Cys)	het	[115]	-
	chr1:216019240-216019240	USH2A c.8981G>A p.(Trp2994Ter)	het	[211]	-
	chr1:216173905-216173905	USH2A c.6326-1G>A	het	novel	-
**12000202+**	chr11:76919517-76919517	MYO7A c.5899C>T p.(Arg1967Ter)	het	novel	-
	chr11:76869378-76869378	MYO7A c.905G>A p.(Arg302His)	het	[212]	minimal effect [213]
	chr11:76853873-76853873	MYO7A c.132+5G>A	het	novel	-
	chr11:76871263-76871263	MYO7A c.1135G>A p.(Gly379Arg)	het	novel	-
**13012581+**	chr1:215963510-215963510	USH2A c.10073G>A p.(Cys3358Tyr)	het	[115, 132, 152, 153, 155]	-
	chr1:215940074-215940074	USH2A c.10996T>G p.(Cys3666Gly)	het	novel	-
**13011433+**	chr1:216420214-216420214	USH2A c.2522C>A p.(Ser841Tyr)	het	[132, 214]	-
	chr1:215853690-215853690	USH2A c.12095G>T p.(Gly4032Val)	het	novel	-
	chr1:216243517-216243517	USH2A c.5975A>G p.(Tyr1992Cys)	het	[132, 152]	-
	chr1:216173760-216173760	USH2A c.6470delG	het	novel	-
**12013066+**	chr1:216040454-216040454	USH2A c.8740C>T p.(Arg2914Ter)	het	[152]	-
	chr1:216380743-216380744	USH2A c.3187_3188delCA	het	[134]	-
**12006582**	chr11:76900513-76900513	MYO7A c.3628A>T p.(Lys1210Ter)	het	novel	-
	chr11:76916643-76916643	MYO7A c.5617C>T p.(Arg1873Trp)	het	[215, 216]	-

Supp. Table S5. Genetic variants reported to referring clinicians, including causative, likely causative, and variants of unknown clinical significance.

MCGM ID	Genomic loci *hg19*	Variant details *specified transcripts, Table S1*	Zygosity	Supporting evidence from literature	
				Disease segregation	Functional
**11012656**	chr1:216495311-216495311	USH2A c.1558delT	hom	novel	-
**13005697**	chr1:216420214-216420214	USH2A c.2522C>A p.(Ser841Tyr)	het	[132, 214]	-
	chr1:215847632-215847632	USH2A c.13621C>T p.(Gln4541Ter)	het	[115]	-
	chr1:215901725-215901725	USH2A c.11713C>T p.(Arg3905Cys)	het	novel	-
**13013364**	chr1:216062399-216062399	USH2A c.7595-3C>G	het	[105-107]	[107]
	chr1:216420075-216420075	USH2A c.2661C>G p.(Tyr887Ter)	het	novel	-
**13001832**	chr1:216420460-216420460	USH2A c.2276G>T p.(Cys759Phe)	hom	[128-132]	-
**13002679**	chr3:150690398-150690398	CLRN1 c.98G>A p.(Trp33Ter)	het	novel	-
	chr3:150645894-150645894	CLRN1 c.567T>G p.(Tyr189Ter)	het	[108, 109]	-
**10002008**+	chr11:76903286-76903286	MYO7A c.4115T>G p.(Val1372Gly)	het	novel	-
	chr11:76919822-76919822	MYO7A c.6025delG	het	[216, 217]	-
**12006031**+	chr1:215972365-215972365	USH2A c.9842G>T p.(Cys3281Phe)	het	[115]	-
	chr1:216420460-216420460	USH2A c.2276G>T p.(Cys759Phe)	het	[128-132]	-
	chr1:215848679-215848679	USH2A c.12574C>T p.(Arg4192Cys)	het	p.Arg4192His [152, 153]	-
	chr1:216373196-216373196	USH2A c.3584G>T p.(Cys1195Phe)	het	novel	-
	chr1:216420480-216420480	USH2A c.2256T>C p.(His752His)	het	[128, 130, 131]	-
**12015458**	chr9:117169063-117169063	DFNB31 c.1808G>T p.(Gly603Val)	het	novel	-
	chr9:117170303-117170303	DFNB31 c.1627-5T>A	het	novel	-
**13008753**	chr1:216270538-216270538	USH2A c.4645C>T p.(Arg1549Ter)	het	[132]	-
	chr1:216380775-216380775	USH2A c.3158-2A>G	het	novel	-
**13002780**+	chr1:216420437-216420437	USH2A c.2299delG	hom	[27, 115, 133-136]	-
**12015030**+	chr1:216369894-216369894	USH2A c.4251+1G>A	het	novel	-
	chr1:216420437-216420437	USH2A c.2299delG	het	[27, 115, 133-136]	-
**12008422**	chr1:216173784-216173784	USH2A c.6446C>A p.(Pro2149Gln)	hom	novel	-

Supp. Table S5. Genetic variants reported to referring clinicians, including causative, likely causative, and variants of unknown clinical significance.

MCGM ID	Genomic loci *hg19*	Variant details *specified transcripts, Table S1*	Zygosity	Supporting evidence from literature	
				Disease segregation	Functional
**13008826**	chr1:216498866-216498867	USH2A c.920_923dupGCCA	het	[218, 219]	-
	chr1:215901562-215901563	USH2A c.11875_11876delCA	het	[220]	-
**12012730**	chr1:216420214-216420214	USH2A c.2522C>A p.(Ser841Tyr)	het	[132, 214]	-
	chr1:215822102-215822102	USH2A c.14350G>T p.(Glu4784Ter)	het	novel	-
	chr1:215823989-215823989	USH2A c.14288G>A p.(Gly4763Glu)	het	p.(Gly4763Arg) [152]	-
**12007398**	chr1:94480243-94480243	ABCA4 c.5316G>A p.(Trp1772Ter)	het	[49]	-
	chr1:94471055-94471055	ABCA4 c.6089G>A p.(Arg2030Gln)	het	[48, 191, 221]	-
**12013349**[+]	chr11:17542412-17542412	USH1C c.1210+5G>A	het	novel	c.1210+6T>G [107]
	chr11:17548769-17548769	USH1C c.496+1G>A	het	[115, 222]	-
**13009855**	chr1:216420437-216420437	USH2A c.2299delG	hom	[27, 115, 133-136]	-
**13000951**	chr10:73461778-73461778	CDH23 c.2398-1G>T	het	novel	-
	chr10:73565598-73565598	CDH23 c.7908C>G p.(Tyr2636Ter)	het	novel	-

Novel, at the time analysis the reported mutation was determined to not have been previously reported in the literature as a cause of inherited retinal disease, or to have been reported as a normal polymorphism. [+], additional familial segregation analysis has been requested and undertaken.

Supporting *functional* evidence includes biochemical assays for variants of interest, and detailed computational modeling and/or evidence-based discussion of the impact the variant of interest will have on protein structure and/or function.

1. Michaelides M, Aligianis IA, Ainsworth JR, Good P, Mollon JD, Maher ER, et al. Progressive cone dystrophy associated with mutation in CNGB3. Invest Ophthalmol Vis Sci. 2004;45: 1975-82.
2. Kohl S, Varsanyi B, Antunes GA, Baumann B, Hoyng CB, Jagle H, et al. CNGB3 mutations account for 50% of all cases with autosomal recessive achromatopsia. Eur J Hum Genet. 2005;13: 302-8.
3. Littink KW, Koenekoop RK, van den Born LI, Collin RW, Moruz L, Veltman JA, et al. Homozygosity mapping in patients with cone-rod dystrophy: novel mutations and clinical characterizations. Invest Ophthalmol Vis Sci. 2010;51: 5943-51.

Supp. Table S5. Genetic variants reported to referring clinicians, including causative, likely causative, and variants of unknown clinical significance.

4. Wang X, Wang H, Sun V, Tuan HF, Keser V, Wang K, et al. Comprehensive molecular diagnosis of 179 Leber congenital amaurosis and juvenile retinitis pigmentosa patients by targeted next generation sequencing. J Med Genet. 2013;50: 674-88.
5. Fathinajafabadi A, Perez-Jimenez E, Riera M, Knecht E, Gonzalez-Duarte R. CERKL, a retinal disease gene, encodes an mRNA-binding protein that localizes in compact and untranslated mRNPs associated with microtubules. PLoS One. 2014;9: e87898.
6. Reuter P, Koeppen K, Ladewig T, Kohl S, Baumann B, Wissinger B. Mutations in CNGA3 impair trafficking or function of cone cyclic nucleotide-gated channels, resulting in achromatopsia. Hum Mutat. 2008;29: 1228-36.
7. Kelsell RE, Gregory-Evans K, Payne AM, Perrault I, Kaplan J, Yang RB, et al. Mutations in the retinal guanylate cyclase (RETGC-1) gene in dominant cone-rod dystrophy. Hum Mol Genet. 1998;7: 1179-84.
8. Payne AM, Morris AG, Downes SM, Johnson S, Bird AC, Moore AT, et al. Clustering and frequency of mutations in the retinal guanylate cyclase (GUCY2D) gene in patients with dominant cone-rod dystrophies. J Med Genet. 2001;38: 611-4.
9. Wilkie SE, Newbold RJ, Deery E, Walker CE, Stinton I, Ramamurthy V, et al. Functional characterization of missense mutations at codon 838 in retinal guanylate cyclase correlates with disease severity in patients with autosomal dominant cone-rod dystrophy. Hum Mol Genet. 2000;9: 3065-73.
10. Thiadens AA, Phan TM, Zekveld-Vroon RC, Leroy BP, van den Born LI, Hoyng CB, et al. Clinical course, genetic etiology, and visual outcome in cone and cone-rod dystrophy. Ophthalmology. 2012;119: 819-26.
11. Allikmets R, Shroyer NF, Singh N, Seddon JM, Lewis RA, Bernstein PS, et al. Mutation of the Stargardt disease gene (ABCR) in age-related macular degeneration. Science. 1997;277: 1805-7.
12. Shroyer NF, Lewis RA, Yatsenko AN, Wensel TG, Lupski JR. Cosegregation and functional analysis of mutant ABCR (ABCA4) alleles in families that manifest both Stargardt disease and age-related macular degeneration. Hum Mol Genet. 2001;10: 2671-8.
13. Biswas EE, Biswas SB. The C-terminal nucleotide binding domain of the human retinal ABCR protein is an adenosine triphosphatase. Biochemistry. 2000;39: 15879-86.
14. Kohl S, Marx T, Giddings I, Jagle H, Jacobson SG, Apfelstedt-Sylla E, et al. Total colourblindness is caused by mutations in the gene encoding the alpha-subunit of the cone photoreceptor cGMP-gated cation channel. Nat Genet. 1998;19: 257-9.
15. Varsanyi B, Wissinger B, Kohl S, Koeppen K, Farkas A. Clinical and genetic features of Hungarian achromatopsia patients. Mol Vis. 2005;11: 996-1001.
16. Matveev AV, Fitzgerald JB, Xu J, Malykhina AP, Rodgers KK, Ding XQ. The disease-causing mutations in the carboxyl terminus of the cone cyclic nucleotide-gated channel CNGA3 subunit alter the local secondary structure and interfere with the channel active conformational change. Biochemistry. 2010;49: 1628-39.
17. Wissinger B, Gamer D, Jagle H, Giorda R, Marx T, Mayer S, et al. CNGA3 mutations in hereditary cone photoreceptor disorders. Am J Hum Genet. 2001;69: 722-37.
18. Koeppen K, Reuter P, Kohl S, Baumann B, Ladewig T, Wissinger B. Functional analysis of human CNGA3 mutations associated with colour blindness suggests impaired surface expression of channel mutants A3(R427C) and A3(R563C). Eur J Neurosci. 2008;27: 2391-401.

19. Fahim AT, Khan NW, Zahid S, Schachar IH, Branham K, Kohl S, et al. Diagnostic fundus autofluorescence patterns in achromatopsia. Am J Ophthalmol. 2013;156: 1211-9 e2.
20. Doucette L, Green J, Black C, Schwartzentruber J, Johnson GJ, Galutira D, et al. Molecular genetics of achromatopsia in Newfoundland reveal genetic heterogeneity, founder effects and the first cases of Jalili syndrome in North America. Ophthalmic Genet. 2013;34: 119-29.
21. Hanein S, Perrault I, Olsen P, Lopponen T, Hietala M, Gerber S, et al. Evidence of a founder effect for the RETGC1 (GUCY2D) 2943DelG mutation in Leber congenital amaurosis pedigrees of Finnish origin. Hum Mutat. 2002;20: 322-3.
22. Tucker CL, Ramamurthy V, Pina AL, Loyer M, Dharmaraj S, Li Y, et al. Functional analyses of mutant recessive GUCY2D alleles identified in Leber congenital amaurosis patients: protein domain comparisons and dominant negative effects. Mol Vis. 2004;10: 297-303.
23. Galvin JA, Fishman GA, Stone EM, Koenekoop RK. Clinical phenotypes in carriers of Leber congenital amaurosis mutations. Ophthalmology. 2005;112: 349-56.
24. Ouechtati F, Merdassi A, Bouyacoub Y, Largueche L, Derouiche K, Ouragini H, et al. Clinical and genetic investigation of a large Tunisian family with complete achromatopsia: identification of a new nonsense mutation in GNAT2 gene. J Hum Genet. 2011;56: 22-8.
25. Johnson S, Michaelides M, Aligianis IA, Ainsworth JR, Mollon JD, Maher ER, et al. Achromatopsia caused by novel mutations in both CNGA3 and CNGB3. J Med Genet. 2004;41: e20.
26. Mykytyn K, Nishimura DY, Searby CC, Shastri M, Yen HJ, Beck JS, et al. Identification of the gene (BBS1) most commonly involved in Bardet-Biedl syndrome, a complex human obesity syndrome. Nat Genet. 2002;31: 435-8.
27. O'Sullivan J, Mullaney BG, Bhaskar SS, Dickerson JE, Hall G, O'Grady A, et al. A paradigm shift in the delivery of services for diagnosis of inherited retinal disease. J Med Genet. 2012;49: 322-6.
28. Nishimura DY, Baye LM, Perveen R, Searby CC, Avila-Fernandez A, Pereiro I, et al. Discovery and functional analysis of a retinitis pigmentosa gene, C2ORF71. Am J Hum Genet. 2010;86: 686-95.
29. Cox KF, Kerr NC, Kedrov M, Nishimura D, Jennings BJ, Stone EM, et al. Phenotypic expression of Bardet-Biedl syndrome in patients homozygous for the common M390R mutation in the BBS1 gene. Vision Res. 2012;75: 77-87.
30. Davis RE, Swiderski RE, Rahmouni K, Nishimura DY, Mullins RF, Agassandian K, et al. A knockin mouse model of the Bardet-Biedl syndrome 1 M390R mutation has cilia defects, ventriculomegaly, retinopathy, and obesity. Proc Natl Acad Sci U S A. 2007;104: 19422-7.
31. Stoetzel C, Muller J, Laurier V, Davis EE, Zaghloul NA, Vicaire S, et al. Identification of a novel BBS gene (BBS12) highlights the major role of a vertebrate-specific branch of chaperonin-related proteins in Bardet-Biedl syndrome. Am J Hum Genet. 2007;80: 1-11.
32. Stoetzel C, Laurier V, Davis EE, Muller J, Rix S, Badano JL, et al. BBS10 encodes a vertebrate-specific chaperonin-like protein and is a major BBS locus. Nat Genet. 2006;38: 521-4.
33. Henderson RH, Mackay DS, Li Z, Moradi P, Sergouniotis P, Russell-Eggitt I, et al. Phenotypic variability in patients with retinal dystrophies due to mutations in CRB1. Br J Ophthalmol. 2011;95: 811-7.
34. den Hollander AI, Davis J, van der Velde-Visser SD, Zonneveld MN, Pierrottet CO, Koenekoop RK, et al. CRB1 mutation spectrum in inherited retinal dystrophies. Hum Mutat. 2004;24: 355-69.

35. Corton M, Tatu SD, Avila-Fernandez A, Vallespin E, Tapias I, Cantalapiedra D, et al. High frequency of CRB1 mutations as cause of Early-Onset Retinal Dystrophies in the Spanish population. Orphanet J Rare Dis. 2013;8: 20.
36. den Hollander AI, ten Brink JB, de Kok YJ, van Soest S, van den Born LI, van Driel MA, et al. Mutations in a human homologue of Drosophila crumbs cause retinitis pigmentosa (RP12). Nat Genet. 1999;23: 217-21.
37. Riveiro-Alvarez R, Vallespin E, Wilke R, Garcia-Sandoval B, Cantalapiedra D, Aguirre-Lamban J, et al. Molecular analysis of ABCA4 and CRB1 genes in a Spanish family segregating both Stargardt disease and autosomal recessive retinitis pigmentosa. Mol Vis. 2008;14: 262-7.
38. Clark GR, Crowe P, Muszynska D, O'Prey D, O'Neill J, Alexander S, et al. Development of a diagnostic genetic test for simplex and autosomal recessive retinitis pigmentosa. Ophthalmology. 2010;117: 2169-77 e3.
39. Simpson DA, Clark GR, Alexander S, Silvestri G, Willoughby CE. Molecular diagnosis for heterogeneous genetic diseases with targeted high-throughput DNA sequencing applied to retinitis pigmentosa. J Med Genet. 2011;48: 145-51.
40. Aguirre-Lamban J, Riveiro-Alvarez R, Garcia-Hoyos M, Cantalapiedra D, Avila-Fernandez A, Villaverde-Montero C, et al. Comparison of high-resolution melting analysis with denaturing high-performance liquid chromatography for mutation scanning in the ABCA4 gene. Invest Ophthalmol Vis Sci. 2010;51: 2615-9.
41. Stenirri S, Battistella S, Soriani N, Bernal S, Baiget M, Ferrari M, et al. Molecular scanning of the ABCA4 gene in Spanish patients with retinitis pigmentosa and Stargardt disease: identification of novel mutations. Eur J Ophthalmol. 2007;17: 749-54.
42. Swain PK, Chen S, Wang QL, Affatigato LM, Coats CL, Brady KD, et al. Mutations in the cone-rod homeobox gene are associated with the cone-rod dystrophy photoreceptor degeneration. Neuron. 1997;19: 1329-36.
43. Mitton KP, Swain PK, Chen S, Xu S, Zack DJ, Swaroop A. The leucine zipper of NRL interacts with the CRX homeodomain. A possible mechanism of transcriptional synergy in rhodopsin regulation. J Biol Chem. 2000;275: 29794-9.
44. Webster AR, Heon E, Lotery AJ, Vandenburgh K, Casavant TL, Oh KT, et al. An analysis of allelic variation in the ABCA4 gene. Invest Ophthalmol Vis Sci. 2001;42: 1179-89.
45. Cideciyan AV, Swider M, Aleman TS, Tsybovsky Y, Schwartz SB, Windsor EA, et al. ABCA4 disease progression and a proposed strategy for gene therapy. Hum Mol Genet. 2009;18: 931-41.
46. September AV, Vorster AA, Ramesar RS, Greenberg LJ. Mutation spectrum and founder chromosomes for the ABCA4 gene in South African patients with Stargardt disease. Invest Ophthalmol Vis Sci. 2004;45: 1705-11.
47. Pras E, Abu A, Rotenstreich Y, Avni I, Reish O, Morad Y, et al. Cone-rod dystrophy and a frameshift mutation in the PROM1 gene. Mol Vis. 2009;15: 1709-16.
48. Lewis RA, Shroyer NF, Singh N, Allikmets R, Hutchinson A, Li Y, et al. Genotype/Phenotype analysis of a photoreceptor-specific ATP-binding cassette transporter gene, ABCR, in Stargardt disease. Am J Hum Genet. 1999;64: 422-34.
49. Briggs CE, Rucinski D, Rosenfeld PJ, Hirose T, Berson EL, Dryja TP. Mutations in ABCR (ABCA4) in patients with Stargardt macular degeneration or cone-rod degeneration. Investigative Ophthalmology & Visual Science. 2001;42: 2229-36.
50. Sun H, Smallwood PM, Nathans J. Biochemical defects in ABCR protein variants associated with human retinopathies. Nat Genet. 2000;26: 242-6.
51. Kitiratschky VBD, Grau T, Bernd A, Zrenner E, Jagle H, Renner AB, et al. ABCA4 gene analysis in patients with autosomal recessive cone and cone rod dystrophies. Eur J Hum Genet. 2008;16: 812-9.

52. Maugeri A, Klevering BJ, Rohrschneider K, Blankenagel A, Brunner HG, Deutman AF, et al. Mutations in the ABCA4 (ABCR) gene are the major cause of autosomal recessive cone-rod dystrophy. Am J Hum Genet. 2000;67: 960-6.
53. Khan MI, Ajmal M, Micheal S, Azam M, Hussain A, Shahzad A, et al. Homozygosity mapping identifies genetic defects in four consanguineous families with retinal dystrophy from Pakistan. Clin Genet. 2013;84: 290-3.
54. Shanks ME, Downes SM, Copley RR, Lise S, Broxholme J, Hudspith KA, et al. Next-generation sequencing (NGS) as a diagnostic tool for retinal degeneration reveals a much higher detection rate in early-onset disease. Eur J Hum Genet. 2013;21: 274-80.
55. Tory K, Lacoste T, Burglen L, Moriniere V, Boddaert N, Macher MA, et al. High NPHP1 and NPHP6 mutation rate in patients with Joubert syndrome and nephronophthisis: potential epistatic effect of NPHP6 and AHI1 mutations in patients with NPHP1 mutations. J Am Soc Nephrol. 2007;18: 1566-75.
56. Wiszniewski W, Lewis RA, Stockton DW, Peng J, Mardon G, Chen R, et al. Potential involvement of more than one locus in trait manifestation for individuals with Leber congenital amaurosis. Hum Genet. 2011;129: 319-27.
57. Liu C, Varnum MD. Functional consequences of progressive cone dystrophy-associated mutations in the human cone photoreceptor cyclic nucleotide-gated channel CNGA3 subunit. Am J Physiol Cell Physiol. 2005;289: C187-98.
58. Breuer DK, Yashar BM, Filippova E, Hiriyanna S, Lyons RH, Mears AJ, et al. A comprehensive mutation analysis of RP2 and RPGR in a North American cohort of families with X-linked retinitis pigmentosa. Am J Hum Genet. 2002;70: 1545-54.
59. Nikopoulos K, Venselaar H, Collin RW, Riveiro-Alvarez R, Boonstra FN, Hooymans JM, et al. Overview of the mutation spectrum in familial exudative vitreoretinopathy and Norrie disease with identification of 21 novel variants in FZD4, LRP5, and NDP. Hum Mutat. 2010;31: 656-66.
60. Kondo H, Hayashi H, Oshima K, Tahira T, Hayashi K. Frizzled 4 gene (FZD4) mutations in patients with familial exudative vitreoretinopathy with variable expressivity. Br J Ophthalmol. 2003;87: 1291-5.
61. Toomes C, Bottomley HM, Scott S, Mackey DA, Craig JE, Appukuttan B, et al. Spectrum and frequency of FZD4 mutations in familial exudative vitreoretinopathy. Invest Ophthalmol Vis Sci. 2004;45: 2083-90.
62. Otto EA, Ramaswami G, Janssen S, Chaki M, Allen SJ, Zhou W, et al. Mutation analysis of 18 nephronophthisis associated ciliopathy disease genes using a DNA pooling and next generation sequencing strategy. J Med Genet. 2011;48: 105-16.
63. Hanein S, Perrault I, Gerber S, Tanguy G, Barbet F, Ducroq D, et al. Leber congenital amaurosis: comprehensive survey of the genetic heterogeneity, refinement of the clinical definition, and genotype-phenotype correlations as a strategy for molecular diagnosis. Hum Mutat. 2004;23: 306-17.
64. Hughes AE, Meng W, Lotery AJ, Bradley DT. A novel GUCY2D mutation, V933A, causes central areolar choroidal dystrophy. Invest Ophthalmol Vis Sci. 2012;53: 4748-53.
65. den Hollander AI, Heckenlively JR, van den Born LI, de Kok YJ, van der Velde-Visser SD, Kellner U, et al. Leber congenital amaurosis and retinitis pigmentosa with Coats-like exudative vasculopathy are associated with mutations in the crumbs homologue 1 (CRB1) gene. Am J Hum Genet. 2001;69: 198-203.
66. Yzer S, Hollander AI, Lopez I, Pott JW, de Faber JT, Cremers FP, et al. Ocular and extra-ocular features of patients with Leber congenital amaurosis and mutations in CEP290. Mol Vis. 2012;18: 412-25.
67. Perrault I, Delphin N, Hanein S, Gerber S, Dufier JL, Roche O, et al. Spectrum of NPHP6/CEP290 mutations in Leber congenital amaurosis and delineation of the associated phenotype. Hum Mutat. 2007;28: 416.

68. McKibbin M, Ali M, Mohamed MD, Booth AP, Bishop F, Pal B, et al. Genotype-phenotype correlation for leber congenital amaurosis in Northern Pakistan. Arch Ophthalmol. 2010;128: 107-13.
69. Coppieters F, Casteels I, Meire F, De Jaegere S, Hooghe S, van Regemorter N, et al. Genetic screening of LCA in Belgium: predominance of CEP290 and identification of potential modifier alleles in AHI1 of CEP290-related phenotypes. Hum Mutat. 2010;31: E1709-66.
70. den Hollander AI, Koenekoop RK, Yzer S, Lopez I, Arends ML, Voesenek KEJ, et al. Mutations in the CEP290 (NPHP6) gene are a frequent cause of leber congenital amaurosis. American Journal of Human Genetics. 2006;79: 556-61.
71. Ramamurthy V, Roberts M, van den Akker F, Niemi G, Reh TA, Hurley JB. AIPL1, a protein implicated in Leber's congenital amaurosis, interacts with and aids in processing of farnesylated proteins. Proc Natl Acad Sci U S A. 2003;100: 12630-5.
72. Jacobson SG, Cideciyan AV, Aleman TS, Sumaroka A, Roman AJ, Swider M, et al. Human retinal disease from AIPL1 gene mutations: foveal cone loss with minimal macular photoreceptors and rod function remaining. Invest Ophthalmol Vis Sci. 2011;52: 70-9.
73. Jacobson SG, Aleman TS, Cideciyan AV, Sumaroka A, Schwartz SB, Windsor EA, et al. Identifying photoreceptors in blind eyes caused by RPE65 mutations: Prerequisite for human gene therapy success. Proc Natl Acad Sci U S A. 2005;102: 6177-82.
74. Bereta G, Kiser PD, Golczak M, Sun W, Heon E, Saperstein DA, et al. Impact of retinal disease-associated RPE65 mutations on retinoid isomerization. Biochemistry. 2008;47: 9856-65.
75. Seong MW, Kim SY, Yu YS, Hwang JM, Kim JY, Park SS. Molecular characterization of Leber congenital amaurosis in Koreans. Mol Vis. 2008;14: 1429-36.
76. Roepman R, Letteboer SJ, Arts HH, van Beersum SE, Lu X, Krieger E, et al. Interaction of nephrocystin-4 and RPGRIP1 is disrupted by nephronophthisis or Leber congenital amaurosis-associated mutations. Proc Natl Acad Sci U S A. 2005;102: 18520-5.
77. Koenekoop RK, Fishman GA, Iannaccone A, Ezzeldin H, Ciccarelli ML, Baldi A, et al. Electroretinographic abnormalities in parents of patients with Leber congenital amaurosis who have heterozygous GUCY2D mutations. Arch Ophthalmol. 2002;120: 1325-30.
78. Pasadhika S, Fishman GA, Stone EM, Lindeman M, Zelkha R, Lopez I, et al. Differential macular morphology in patients with RPE65-, CEP290-, GUCY2D-, and AIPL1-related Leber congenital amaurosis. Invest Ophthalmol Vis Sci. 2010;51: 2608-14.
79. Dryja TP, Adams SM, Grimsby JL, McGee TL, Hong DH, Li T, et al. Null RPGRIP1 alleles in patients with Leber congenital amaurosis. Am J Hum Genet. 2001;68: 1295-8.
80. Dharmaraj SR, Silva ER, Pina AL, Li YY, Yang JM, Carter CR, et al. Mutational analysis and clinical correlation in Leber congenital amaurosis. Ophthalmic Genet. 2000;21: 135-50.
81. Silva E, Dharmaraj S, Li YY, Pina AL, Carter RC, Loyer M, et al. A missense mutation in GUCY2D acts as a genetic modifier in RPE65-related Leber Congenital Amaurosis. Ophthalmic Genet. 2004;25: 205-17.
82. Thompson DA, Gyurus P, Fleischer LL, Bingham EL, McHenry CL, Apfelstedt-Sylla E, et al. Genetics and phenotypes of RPE65 mutations in inherited retinal degeneration. Invest Ophthalmol Vis Sci. 2000;41: 4293-9.
83. Rivolta C, Berson EL, Dryja TP. Dominant Leber congenital amaurosis, cone-rod degeneration, and retinitis pigmentosa caused by mutant versions of the transcription factor CRX. Hum Mutat. 2001;18: 488-98.

84. Rivolta C, Peck NE, Fulton AB, Fishman GA, Berson EL, Dryja TP. Novel frameshift mutations in CRX associated with Leber congenital amaurosis. Hum Mutat. 2001;18: 550-1.
85. Levy G, Levi-Acobas F, Blanchard S, Gerber S, Larget-Piet D, Chenal V, et al. Myosin VIIA gene: heterogeneity of the mutations responsible for Usher syndrome type IB. Hum Mol Genet. 1997;6: 111-6.
86. Jacobson SG, Aleman TS, Sumaroka A, Cideciyan AV, Roman AJ, Windsor EA, et al. Disease boundaries in the retina of patients with Usher syndrome caused by MYO7A gene mutations. Invest Ophthalmol Vis Sci. 2009;50: 1886-94.
87. Chaki M, Hoefele J, Allen SJ, Ramaswami G, Janssen S, Bergmann C, et al. Genotype-phenotype correlation in 440 patients with NPHP-related ciliopathies. Kidney Int. 2011;80: 1239-45.
88. Vallespin E, Cantalapiedra D, Riveiro-Alvarez R, Aguirre-Lamban J, Avila-Fernandez A, Martinez MA, et al. Human gene mutations. Gene symbol: CRB1. Disease: late onset retinitis pigmentosa. Hum Genet. 2007;122: 212.
89. Simovich MJ, Miller B, Ezzeldin H, Kirkland BT, McLeod G, Fulmer C, et al. Four novel mutations in the RPE65 gene in patients with Leber congenital amaurosis. Hum Mutat. 2001;18: 164.
90. Redmond TM, Poliakov E, Yu S, Tsai JY, Lu Z, Gentleman S. Mutation of key residues of RPE65 abolishes its enzymatic role as isomerohydrolase in the visual cycle. Proc Natl Acad Sci U S A. 2005;102: 13658-63.
91. Philp AR, Jin M, Li S, Schindler EI, Iannaccone A, Lam BL, et al. Predicting the pathogenicity of RPE65 mutations. Hum Mutat. 2009;30: 1183-8.
92. Thompson DA, Janecke AR, Lange J, Feathers KL, Hubner CA, McHenry CL, et al. Retinal degeneration associated with RDH12 mutations results from decreased 11-cis retinal synthesis due to disruption of the visual cycle. Hum Mol Genet. 2005;14: 3865-75.
93. Jacobson SG, Cideciyan AV, Aleman TS, Sumaroka A, Schwartz SB, Windsor EA, et al. RDH12 and RPE65, visual cycle genes causing leber congenital amaurosis, differ in disease expression. Invest Ophthalmol Vis Sci. 2007;48: 332-8.
94. Littink KW, Pott JW, Collin RW, Kroes HY, Verheij JB, Blokland EA, et al. A novel nonsense mutation in CEP290 induces exon skipping and leads to a relatively mild retinal phenotype. Invest Ophthalmol Vis Sci. 2010;51: 3646-52.
95. Sohocki MM, Bowne SJ, Sullivan LS, Blackshaw S, Cepko CL, Payne AM, et al. Mutations in a new photoreceptor-pineal gene on 17p cause Leber congenital amaurosis. Nat Genet. 2000;24: 79-83.
96. van der Spuy J, Cheetham ME. The Leber congenital amaurosis protein AIPL1 modulates the nuclear translocation of NUB1 and suppresses inclusion formation by NUB1 fragments. J Biol Chem. 2004;279: 48038-47.
97. Lee SA, Belyaeva OV, Popov IK, Kedishvili NY. Overproduction of bioactive retinoic acid in cells expressing disease-associated mutants of retinol dehydrogenase 12. J Biol Chem. 2007;282: 35621-8.
98. Jacobson SG, Cideciyan AV, Peshenko IV, Sumaroka A, Olshevskaya EV, Cao L, et al. Determining consequences of retinal membrane guanylyl cyclase (RetGC1) deficiency in human Leber congenital amaurosis en route to therapy: residual cone-photoreceptor vision correlates with biochemical properties of the mutants. Hum Mol Genet. 2013;22: 168-83.
99. Harville HM, Held S, Diaz-Font A, Davis EE, Diplas BH, Lewis RA, et al. Identification of 11 novel mutations in eight BBS genes by high-resolution homozygosity mapping. J Med Genet. 2010;47: 262-7.
100. Ajmal M, Khan MI, Neveling K, Khan YM, Ali SH, Ahmed W, et al. Novel mutations in RDH5 cause fundus albipunctatus in two consanguineous Pakistani families. Mol Vis. 2012;18: 1558-71.
101. Benaglio P, McGee TL, Capelli LP, Harper S, Berson EL, Rivolta C. Next generation sequencing of pooled samples reveals new SNRNP200 mutations associated with retinitis pigmentosa. Hum Mutat. 2011;32: E2246-58.

102. Chen LJ, Lai TY, Tam PO, Chiang SW, Zhang X, Lam S, et al. Compound heterozygosity of two novel truncation mutations in RP1 causing autosomal recessive retinitis pigmentosa. Invest Ophthalmol Vis Sci. 2010;51: 2236-42.
103. Tschernutter M, Jenkins SA, Waseem NH, Saihan Z, Holder GE, Bird AC, et al. Clinical characterisation of a family with retinal dystrophy caused by mutation in the Mertk gene. Br J Ophthalmol. 2006;90: 718-23.
104. Ali M, Ramprasad VL, Soumittra N, Mohamed MD, Jafri H, Rashid Y, et al. A missense mutation in the nuclear localization signal sequence of CERKL (p.R106S) causes autosomal recessive retinal degeneration. Mol Vis. 2008;14: 1960-4.
105. Baux D, Larrieu L, Blanchet C, Hamel C, Ben Salah S, Vielle A, et al. Molecular and in silico analyses of the full-length isoform of usherin identify new pathogenic alleles in Usher type II patients. Hum Mutat. 2007;28: 781-9.
106. Baux D, Faugere V, Larrieu L, Le Guedard-Mereuze S, Hamroun D, Beroud C, et al. UMD-USHbases: a comprehensive set of databases to record and analyse pathogenic mutations and unclassified variants in seven Usher syndrome causing genes. Hum Mutat. 2008;29: E76-87.
107. Le Guedard-Mereuze S, Vache C, Baux D, Faugere V, Larrieu L, Abadie C, et al. Ex vivo splicing assays of mutations at noncanonical positions of splice sites in USHER genes. Hum Mutat. 2010;31: 347-55.
108. Fields RR, Zhou G, Huang D, Davis JR, Moller C, Jacobson SG, et al. Usher syndrome type III: revised genomic structure of the USH3 gene and identification of novel mutations. Am J Hum Genet. 2002;71: 607-17.
109. Joensuu T, Hamalainen R, Yuan B, Johnson C, Tegelberg S, Gasparini P, et al. Mutations in a novel gene with transmembrane domains underlie Usher syndrome type 3. Am J Hum Genet. 2001;69: 673-84.
110. Isosomppi J, Vastinsalo H, Geller SF, Heon E, Flannery JG, Sankila EM. Disease-causing mutations in the CLRN1 gene alter normal CLRN1 protein trafficking to the plasma membrane. Mol Vis. 2009;15: 1806-18.
111. Haider NB, Jacobson SG, Cideciyan AV, Swiderski R, Streb LM, Searby C, et al. Mutation of a nuclear receptor gene, NR2E3, causes enhanced S cone syndrome, a disorder of retinal cell fate. Nat Genet. 2000;24: 127-31.
112. Sharon D, Sandberg MA, Caruso RC, Berson EL, Dryja TP. Shared mutations in NR2E3 in enhanced S-cone syndrome, Goldmann-Favre syndrome, and many cases of clumped pigmentary retinal degeneration. Arch Ophthalmol. 2003;121: 1316-23.
113. Wright AF, Reddick AC, Schwartz SB, Ferguson JS, Aleman TS, Kellner U, et al. Mutation analysis of NR2E3 and NRL genes in Enhanced S Cone Syndrome. Hum Mutat. 2004;24: 439.
114. Bernal S, Solans T, Gamundi MJ, Hernan I, de Jorge L, Carballo M, et al. Analysis of the involvement of the NR2E3 gene in autosomal recessive retinal dystrophies. Clin Genet. 2008;73: 360-6.
115. Le Quesne Stabej P, Saihan Z, Rangesh N, Steele-Stallard HB, Ambrose J, Coffey A, et al. Comprehensive sequence analysis of nine Usher syndrome genes in the UK National Collaborative Usher Study. J Med Genet. 2012;49: 27-36.
116. Sharon D, Sandberg MA, Rabe VW, Stillberger M, Dryja TP, Berson EL. RP2 and RPGR mutations and clinical correlations in patients with X-linked retinitis pigmentosa. Am J Hum Genet. 2003;73: 1131-46.
117. Prokisch H, Hartig M, Hellinger R, Meitinger T, Rosenberg T. A population-based epidemiological and genetic study of X-linked retinitis pigmentosa. Invest Ophthalmol Vis Sci. 2007;48: 4012-8.
118. Pierce EA, Quinn T, Meehan T, McGee TL, Berson EL, Dryja TP. Mutations in a gene encoding a new oxygen-regulated photoreceptor protein cause dominant retinitis pigmentosa. Nat Genet. 1999;22: 248-54.
119. Schwartz SB, Aleman TS, Cideciyan AV, Swaroop A, Jacobson SG, Stone EM. De novo mutation in the RP1 gene (Arg677ter) associated with retinitis pigmentosa. Invest Ophthalmol Vis Sci. 2003;44: 3593-7.

Supp. Table S5. Genetic variants reported to referring clinicians, including causative, likely causative, and variants of unknown clinical significance.

120. Ramsden SC, O'Grady A, Fletcher T, O'Sullivan J, Hart-Holden N, Barton SJ, et al. A clinical molecular genetic service for United Kingdom families with choroideraemia. Eur J Med Genet. 2013;56: 432-8.
121. Bandah-Rozenfeld D, Mizrahi-Meissonnier L, Farhy C, Obolensky A, Chowers I, Pe'er J, et al. Homozygosity mapping reveals null mutations in FAM161A as a cause of autosomal-recessive retinitis pigmentosa. Am J Hum Genet. 2010;87: 382-91.
122. Langmann T, Di Gioia SA, Rau I, Stohr H, Maksimovic NS, Corbo JC, et al. Nonsense mutations in FAM161A cause RP28-associated recessive retinitis pigmentosa. Am J Hum Genet. 2010;87: 376-81.
123. Zhao C, Bellur DL, Lu S, Zhao F, Grassi MA, Bowne SJ, et al. Autosomal-dominant retinitis pigmentosa caused by a mutation in SNRNP200, a gene required for unwinding of U4/U6 snRNAs. Am J Hum Genet. 2009;85: 617-27.
124. Cvackova Z, Mateju D, Stanek D. Retinitis pigmentosa mutations of SNRNP200 enhance cryptic splice-site recognition. Hum Mutat. 2014;35: 308-17.
125. Waseem NH, Vaclavik V, Webster A, Jenkins SA, Bird AC, Bhattacharya SS. Mutations in the gene coding for the pre-mRNA splicing factor, PRPF31, in patients with autosomal dominant retinitis pigmentosa. Invest Ophthalmol Vis Sci. 2007;48: 1330-4.
126. Vithana EN, Abu-Safieh L, Allen MJ, Carey A, Papaioannou M, Chakarova C, et al. A human homolog of yeast pre-mRNA splicing gene, PRP31, underlies autosomal dominant retinitis pigmentosa on chromosome 19q13.4 (RP11). Mol Cell. 2001;8: 375-81.
127. Koenekoop RK, Loyer M, Hand CK, Al Mahdi H, Dembinska O, Beneish R, et al. Novel RPGR mutations with distinct retinitis pigmentosa phenotypes in French-Canadian families. Am J Ophthalmol. 2003;136: 678-87.
128. Rivolta C, Sweklo EA, Berson EL, Dryja TP. Missense mutation in the USH2A gene: association with recessive retinitis pigmentosa without hearing loss. Am J Hum Genet. 2000;66: 1975-8.
129. Rivolta C, Berson EL, Dryja TP. Paternal uniparental heterodisomy with partial isodisomy of chromosome 1 in a patient with retinitis pigmentosa without hearing loss and a missense mutation in the Usher syndrome type II gene USH2A. Arch Ophthalmol. 2002;120: 1566-71.
130. Aller E, Najera C, Millan JM, Oltra JS, Perez-Garrigues H, Vilela C, et al. Genetic analysis of 2299delG and C759F mutations (USH2A) in patients with visual and/or auditory impairments. Eur J Hum Genet. 2004;12: 407-10.
131. Dreyer B, Tranebjaerg L, Rosenberg T, Weston MD, Kimberling WJ, Nilssen O. Identification of novel USH2A mutations: implications for the structure of USH2A protein. Eur J Hum Genet. 2000;8: 500-6.
132. Garcia-Garcia G, Aparisi MJ, Jaijo T, Rodrigo R, Leon AM, Avila-Fernandez A, et al. Mutational screening of the USH2A gene in Spanish USH patients reveals 23 novel pathogenic mutations. Orphanet J Rare Dis. 2011;6: 65.
133. Bernal S, Ayuso C, Antinolo G, Gimenez A, Borrego S, Trujillo MJ, et al. Mutations in USH2A in Spanish patients with autosomal recessive retinitis pigmentosa: high prevalence and phenotypic variation. J Med Genet. 2003;40: e8.
134. Seyedahmadi BJ, Rivolta C, Keene JA, Berson EL, Dryja TP. Comprehensive screening of the USH2A gene in Usher syndrome type II and non-syndromic recessive retinitis pigmentosa. Exp Eye Res. 2004;79: 167-73.
135. Eudy JD, Weston MD, Yao S, Hoover DM, Rehm HL, Ma-Edmonds M, et al. Mutation of a gene encoding a protein with extracellular matrix motifs in Usher syndrome type IIa. Science. 1998;280: 1753-7.
136. Aller E, Larrieu L, Jaijo T, Baux D, Espinos C, Gonzalez-Candelas F, et al. The USH2A c.2299delG mutation: dating its common origin in a Southern European population. Eur J Hum Genet. 2010;18: 788-93.

137. Keen TJ, Inglehearn CF, Lester DH, Bashir R, Jay M, Bird AC, et al. Autosomal dominant retinitis pigmentosa: four new mutations in rhodopsin, one of them in the retinal attachment site. Genomics. 1991;11: 199-205.
138. Sancho-Pelluz J, Tosi J, Hsu CW, Lee F, Wolpert K, Tabacaru MR, et al. Mice with a D190N mutation in the gene encoding rhodopsin: a model for human autosomal-dominant retinitis pigmentosa. Mol Med. 2012;18: 549-55.
139. Abd El-Aziz MM, O'Driscoll CA, Kaye RS, Barragan I, El-Ashry MF, Borrego S, et al. Identification of novel mutations in the ortholog of Drosophila eyes shut gene (EYS) causing autosomal recessive retinitis pigmentosa. Invest Ophthalmol Vis Sci. 2010;51: 4266-72.
140. Sun W, Gerth C, Maeda A, Lodowski DT, Van Der Kraak L, Saperstein DA, et al. Novel RDH12 mutations associated with Leber congenital amaurosis and cone-rod dystrophy: biochemical and clinical evaluations. Vision Res. 2007;47: 2055-66.
141. Downes SM, Holder GE, Fitzke FW, Payne AM, Warren MJ, Bhattacharya SS, et al. Autosomal dominant cone and cone-rod dystrophy with mutations in the guanylate cyclase activator 1A gene-encoding guanylate cyclase activating protein-1. Arch Ophthalmol. 2001;119: 96-105.
142. Newbold RJ, Deery EC, Walker CE, Wilkie SE, Srinivasan N, Hunt DM, et al. The destabilization of human GCAP1 by a proline to leucine mutation might cause cone-rod dystrophy. Hum Mol Genet. 2001;10: 47-54.
143. McLaughlin ME, Ehrhart TL, Berson EL, Dryja TP. Mutation spectrum of the gene encoding the beta subunit of rod phosphodiesterase among patients with autosomal recessive retinitis pigmentosa. Proc Natl Acad Sci U S A. 1995;92: 3249-53.
144. Dryja TP, McGee TL, Hahn LB, Cowley GS, Olsson JE, Reichel E, et al. Mutations within the rhodopsin gene in patients with autosomal dominant retinitis pigmentosa. N Engl J Med. 1990;323: 1302-7.
145. Audo I, Manes G, Mohand-Said S, Friedrich A, Lancelot ME, Antonio A, et al. Spectrum of rhodopsin mutations in French autosomal dominant rod-cone dystrophy patients. Invest Ophthalmol Vis Sci. 2010;51: 3687-700.
146. Rakoczy EP, Kiel C, McKeone R, Stricher F, Serrano L. Analysis of disease-linked rhodopsin mutations based on structure, function, and protein stability calculations. J Mol Biol. 2011;405: 584-606.
147. Fujinami K, Zernant J, Chana RK, Wright GA, Tsunoda K, Ozawa Y, et al. ABCA4 gene screening by next-generation sequencing in a British cohort. Invest Ophthalmol Vis Sci. 2013;54: 6662-74.
148. Strom SP, Gao YQ, Martinez A, Ortube C, Chen Z, Nelson SF, et al. Molecular diagnosis of putative Stargardt Disease probands by exome sequencing. BMC Med Genet. 2012;13: 67.
149. Zernant J, Schubert C, Im KM, Burke T, Brown CM, Fishman GA, et al. Analysis of the ABCA4 gene by next-generation sequencing. Invest Ophthalmol Vis Sci. 2011;52: 8479-87.
150. Payne A, Vithana E, Khaliq S, Hameed A, Deller J, Abu-Safieh L, et al. RP1 protein truncating mutations predominate at the RP1 adRP locus. Invest Ophthalmol Vis Sci. 2000;41: 4069-73.
151. van den Hurk JA, van de Pol DJ, Wissinger B, van Driel MA, Hoefsloot LH, de Wijs IJ, et al. Novel types of mutation in the choroideremia (CHM) gene: a full-length L1 insertion and an intronic mutation activating a cryptic exon. Hum Genet. 2003;113: 268-75.
152. McGee TL, Seyedahmadi BJ, Sweeney MO, Dryja TP, Berson EL. Novel mutations in the long isoform of the USH2A gene in patients with Usher syndrome type II or non-syndromic retinitis pigmentosa. J Med Genet. 2010;47: 499-506.

153. Avila-Fernandez A, Cantalapiedra D, Aller E, Vallespin E, Aguirre-Lamban J, Blanco-Kelly F, et al. Mutation analysis of 272 Spanish families affected by autosomal recessive retinitis pigmentosa using a genotyping microarray. Molecular Vision. 2010;16: 2550-8.
154. Jayasundera T, Branham KE, Othman M, Rhoades WR, Karoukis AJ, Khanna H, et al. RP2 phenotype and pathogenetic correlations in X-linked retinitis pigmentosa. Arch Ophthalmol. 2010;128: 915-23.
155. Neveling K, Collin RWJ, Gilissen C, van Huet RAC, Visser L, Kwint MP, et al. Next-Generation Genetic Testing for Retinitis Pigmentosa. Human Mutation. 2012;33: 963-72.
156. Yin J, Brocher J, Fischer U, Winkler C. Mutant Prpf31 causes pre-mRNA splicing defects and rod photoreceptor cell degeneration in a zebrafish model for Retinitis pigmentosa. Mol Neurodegener. 2011;6: 56.
157. Schwahn U, Lenzner S, Dong J, Feil S, Hinzmann B, van Duijnhoven G, et al. Positional cloning of the gene for X-linked retinitis pigmentosa 2. Nat Genet. 1998;19: 327-32.
158. Bartolini F, Bhamidipati A, Thomas S, Schwahn U, Lewis SA, Cowan NJ. Functional overlap between retinitis pigmentosa 2 protein and the tubulin-specific chaperone cofactor C. J Biol Chem. 2002;277: 14629-34.
159. Pennings RJ, Huygen PL, Orten DJ, Wagenaar M, van Aarem A, Kremer H, et al. Evaluation of visual impairment in Usher syndrome 1b and Usher syndrome 2a. Acta Ophthalmol Scand. 2004;82: 131-9.
160. Bonnet C, Grati M, Marlin S, Levilliers J, Hardelin JP, Parodi M, et al. Complete exon sequencing of all known Usher syndrome genes greatly improves molecular diagnosis. Orphanet J Rare Dis. 2011;6: 21.
161. Vache C, Besnard T, Blanchet C, Baux D, Larrieu L, Faugere V, et al. Nasal epithelial cells are a reliable source to study splicing variants in Usher syndrome. Hum Mutat. 2010;31: 734-41.
162. Dryja TP, Hahn LB, Cowley GS, McGee TL, Berson EL. Mutation spectrum of the rhodopsin gene among patients with autosomal dominant retinitis pigmentosa. Proc Natl Acad Sci U S A. 1991;88: 9370-4.
163. Cideciyan AV, Hood DC, Huang Y, Banin E, Li ZY, Stone EM, et al. Disease sequence from mutant rhodopsin allele to rod and cone photoreceptor degeneration in man. Proc Natl Acad Sci U S A. 1998;95: 7103-8.
164. Blanco-Kelly F, Garcia-Hoyos M, Corton M, Avila-Fernandez A, Riveiro-Alvarez R, Gimenez A, et al. Genotyping microarray: mutation screening in Spanish families with autosomal dominant retinitis pigmentosa. Mol Vis. 2012;18: 1478-83.
165. Yan EC, Kazmi MA, De S, Chang BS, Seibert C, Marin EP, et al. Function of extracellular loop 2 in rhodopsin: glutamic acid 181 modulates stability and absorption wavelength of metarhodopsin II. Biochemistry. 2002;41: 3620-7.
166. Weber BH, Vogt G, Pruett RC, Stohr H, Felbor U. Mutations in the tissue inhibitor of metalloproteinases-3 (TIMP3) in patients with Sorsby's fundus dystrophy. Nat Genet. 1994;8: 352-6.
167. Morimura H, Fishman GA, Grover SA, Fulton AB, Berson EL, Dryja TP. Mutations in the RPE65 gene in patients with autosomal recessive retinitis pigmentosa or leber congenital amaurosis. Proc Natl Acad Sci U S A. 1998;95: 3088-93.
168. Song J, Smaoui N, Ayyagari R, Stiles D, Benhamed S, MacDonald IM, et al. High-throughput retina-array for screening 93 genes involved in inherited retinal dystrophy. Invest Ophthalmol Vis Sci. 2011;52: 9053-60.
169. Adato A, Vreugde S, Joensuu T, Avidan N, Hamalainen R, Belenkiy O, et al. USH3A transcripts encode clarin-1, a four-transmembrane-domain protein with a possible role in sensory synapses. Eur J Hum Genet. 2002;10: 339-50.
170. Herrera W, Aleman TS, Cideciyan AV, Roman AJ, Banin E, Ben-Yosef T, et al. Retinal disease in Usher syndrome III caused by mutations in the clarin-1 gene. Invest Ophthalmol Vis Sci. 2008;49: 2651-60.

171. Gregory-Evans K, Kelsell RE, Gregory-Evans CY, Downes SM, Fitzke FW, Holder GE, et al. Autosomal dominant cone-rod retinal dystrophy (CORD6) from heterozygous mutation of GUCY2D, which encodes retinal guanylate cyclase. Ophthalmology. 2000;107: 55-61.
172. Yoshida S, Yamaji Y, Yoshida A, Kuwahara R, Yamamoto K, Kubata T, et al. Novel triple missense mutations of GUCY2D gene in Japanese family with cone-rod dystrophy: possible use of genotyping microarray. Mol Vis. 2006;12: 1558-64.
173. Brancati F, Barrano G, Silhavy JL, Marsh SE, Travaglini L, Bielas SL, et al. CEP290 mutations are frequently identified in the oculo-renal form of Joubert syndrome-related disorders. Am J Hum Genet. 2007;81: 104-13.
174. Vervoort R, Lennon A, Bird AC, Tulloch B, Axton R, Miano MG, et al. Mutational hot spot within a new RPGR exon in X-linked retinitis pigmentosa. Nat Genet. 2000;25: 462-6.
175. Meindl A, Dry K, Herrmann K, Manson F, Ciccodicola A, Edgar A, et al. A gene (RPGR) with homology to the RCC1 guanine nucleotide exchange factor is mutated in X-linked retinitis pigmentosa (RP3). Nat Genet. 1996;13: 35-42.
176. Sohocki MM, Daiger SP, Bowne SJ, Rodriquez JA, Northrup H, Heckenlively JR, et al. Prevalence of mutations causing retinitis pigmentosa and other inherited retinopathies. Hum Mutat. 2001;17: 42-51.
177. Sullivan LS, Bowne SJ, Birch DG, Hughbanks-Wheaton D, Heckenlively JR, Lewis RA, et al. Prevalence of disease-causing mutations in families with autosomal dominant retinitis pigmentosa: A screen of known genes in 200 families. Investigative Ophthalmology & Visual Science. 2006;47: 3052-64.
178. Bujakowska K, Audo I, Mohand-Said S, Lancelot ME, Antonio A, Germain A, et al. CRB1 mutations in inherited retinal dystrophies. Hum Mutat. 2012;33: 306-15.
179. Henderson RH, Williamson KA, Kennedy JS, Webster AR, Holder GE, Robson AG, et al. A rare de novo nonsense mutation in OTX2 causes early onset retinal dystrophy and pituitary dysfunction. Mol Vis. 2009;15: 2442-7.
180. Lotery AJ, Munier FL, Fishman GA, Weleber RG, Jacobson SG, Affatigato LM, et al. Allelic variation in the VMD2 gene in best disease and age-related macular degeneration. Invest Ophthalmol Vis Sci. 2000;41: 1291-6.
181. Hayward C, Shu X, Cideciyan AV, Lennon A, Barran P, Zareparsi S, et al. Mutation in a short-chain collagen gene, CTRP5, results in extracellular deposit formation in late-onset retinal degeneration: a genetic model for age-related macular degeneration. Hum Mol Genet. 2003;12: 2657-67.
182. Mackay DS, Ocaka LA, Borman AD, Sergouniotis PI, Henderson RH, Moradi P, et al. Screening of SPATA7 in patients with Leber congenital amaurosis and severe childhood-onset retinal dystrophy reveals disease-causing mutations. Invest Ophthalmol Vis Sci. 2011;52: 3032-8.
183. Sheffield VC, Fishman GA, Beck JS, Kimura AE, Stone EM. Identification of novel rhodopsin mutations associated with retinitis pigmentosa by GC-clamped denaturing gradient gel electrophoresis. Am J Hum Genet. 1991;49: 699-706.
184. Bukowy-Bieryllo Z, Zietkiewicz E, Loges NT, Wittmer M, Geremek M, Olbrich H, et al. RPGR mutations might cause reduced orientation of respiratory cilia. Pediatr Pulmonol. 2013;48: 352-63.
185. Pelletier V, Jambou M, Delphin N, Zinovieva E, Stum M, Gigarel N, et al. Comprehensive survey of mutations in RP2 and RPGR in patients affected with distinct retinal dystrophies: genotype-phenotype correlations and impact on genetic counseling. Hum Mutat. 2007;28: 81-91.
186. Demirci FY, Radak AL, Rigatti BW, Mah TS, Gorin MB. A presumed missense mutation of RPGR causes abnormal RNA splicing with exon skipping. Am J Ophthalmol. 2004;138: 504-5.

187. Riveiro-Alvarez R, Aguirre-Lamban J, Lopez-Martinez MA, Trujillo-Tiebas MJ, Cantalapiedra D, Vallespin E, et al. Frequency of ABCA4 mutations in 278 Spanish controls: an insight into the prevalence of autosomal recessive Stargardt disease. Br J Ophthalmol. 2009;93: 1359-64.
188. Simonelli F, Testa F, de Crecchio G, Rinaldi E, Hutchinson A, Atkinson A, et al. New ABCR mutations and clinical phenotype in Italian patients with Stargardt disease. Invest Ophthalmol Vis Sci. 2000;41: 892-7.
189. Valverde D, Riveiro-Alvarez R, Bernal S, Jaakson K, Baiget M, Navarro R, et al. Microarray-based mutation analysis of the ABCA4 gene in Spanish patients with Stargardt disease: evidence of a prevalent mutated allele. Mol Vis. 2006;12: 902-8.
190. Barbazetto IA, Yannuzzi NA, Klais CM, Merriam JE, Zernant J, Peiretti E, et al. Pseudo-vitelliform macular detachment and cuticular drusen: exclusion of 6 candidate genes. Ophthalmic Genet. 2007;28: 192-7.
191. Hwang JC, Zernant J, Allikmets R, Barile GR, Chang S, Smith RT. Peripapillary atrophy in Stargardt disease. Retina. 2009;29: 181-6.
192. Chacon-Camacho OF, Camarillo-Blancarte L, Zenteno JC. OCT findings in young asymptomatic subjects carrying familial BEST1 gene mutations. Ophthalmic Genet. 2011;32: 24-30.
193. Marchant D, Yu K, Bigot K, Roche O, Germain A, Bonneau D, et al. New VMD2 gene mutations identified in patients affected by Best vitelliform macular dystrophy. J Med Genet. 2007;44: e70.
194. Burke TR, Tsang SH, Zernant J, Smith RT, Allikmets R. Familial discordance in Stargardt disease. Mol Vis. 2012;18: 227-33.
195. Cella W, Greenstein VC, Zernant-Rajang J, Smith TR, Barile G, Allikmets R, et al. G1961E mutant allele in the Stargardt disease gene ABCA4 causes bull's eye maculopathy. Exp Eye Res. 2009;89: 16-24.
196. Payne AM, Downes SM, Bessant DA, Bird AC, Bhattacharya SS. Founder effect, seen in the British population, of the 172 peripherin/RDS mutation-and further refinement of genetic positioning of the peripherin/RDS gene. Am J Hum Genet. 1998;62: 192-5.
197. Wiszniewski W, Zaremba CM, Yatsenko AN, Jamrich M, Wensel TG, Lewis RA, et al. ABCA4 mutations causing mislocalization are found frequently in patients with severe retinal dystrophies. Hum Mol Genet. 2005;14: 2769-78.
198. Roberts LJ, Nossek CA, Greenberg LJ, Ramesar RS. Stargardt macular dystrophy: common ABCA4 mutations in South Africa--establishment of a rapid genetic test and relating risk to patients. Mol Vis. 2012;18: 280-9.
199. Kinnick TR, Mullins RF, Dev S, Leys M, Mackey DA, Kay CN, et al. Autosomal recessive vitelliform macular dystrophy in a large cohort of vitelliform macular dystrophy patients. Retina. 2011;31: 581-95.
200. Sodi A, Menchini F, Manitto MP, Passerini I, Murro V, Torricelli F, et al. Ocular phenotypes associated with biallelic mutations in BEST1 in Italian patients. Mol Vis. 2011;17: 3078-87.
201. Borman AD, Davidson AE, O'Sullivan J, Thompson DA, Robson AG, De Baere E, et al. Childhood-onset autosomal recessive bestrophinopathy. Arch Ophthalmol. 2011;129: 1088-93.
202. Iannaccone A, Kerr NC, Kinnick TR, Calzada JI, Stone EM. Autosomal recessive best vitelliform macular dystrophy: report of a family and management of early-onset neovascular complications. Arch Ophthalmol. 2011;129: 211-7.
203. Burgess R, Millar ID, Leroy BP, Urquhart JE, Fearon IM, De Baere E, et al. Biallelic mutation of BEST1 causes a distinct retinopathy in humans. Am J Hum Genet. 2008;82: 19-31.

204. Kramer F, White K, Pauleikhoff D, Gehrig A, Passmore L, Rivera A, et al. Mutations in the VMD2 gene are associated with juvenile-onset vitelliform macular dystrophy (Best disease) and adult vitelliform macular dystrophy but not age-related macular degeneration. Eur J Hum Genet. 2000;8: 286-92.
205. Schatz P, Klar J, Andreasson S, Ponjavic V, Dahl N. Variant phenotype of Best vitelliform macular dystrophy associated with compound heterozygous mutations in VMD2. Ophthalmic Genet. 2006;27: 51-6.
206. Davidson AE, Millar ID, Burgess-Mullan R, Maher GJ, Urquhart JE, Brown PD, et al. Functional characterization of bestrophin-1 missense mutations associated with autosomal recessive bestrophinopathy. Invest Ophthalmol Vis Sci. 2011;52: 3730-6.
207. Thiagalingam S, McGee TL, Weleber RG, Sandberg MA, Trzupek KM, Berson EL, et al. Novel mutations in the KCNV2 gene in patients with cone dystrophy and a supernormal rod electroretinogram. Ophthalmic Genet. 2007;28: 135-42.
208. Wissinger B, Dangel S, Jagle H, Hansen L, Baumann B, Rudolph G, et al. Cone dystrophy with supernormal rod response is strictly associated with mutations in KCNV2. Invest Ophthalmol Vis Sci. 2008;49: 751-7.
209. Friedburg C, Wissinger B, Schambeck M, Bonin M, Kohl S, Lorenz B. Long-term follow-up of the human phenotype in three siblings with cone dystrophy associated with a homozygous p.G461R mutation of KCNV2. Invest Ophthalmol Vis Sci. 2011;52: 8621-9.
210. Rivera A, White K, Stohr H, Steiner K, Hemmrich N, Grimm T, et al. A comprehensive survey of sequence variation in the ABCA4 (ABCR) gene in Stargardt disease and age-related macular degeneration. Am J Hum Genet. 2000;67: 800-13.
211. Yan D, Ouyang X, Patterson DM, Du LL, Jacobson SG, Liu XZ. Mutation analysis in the long isoform of USH2A in American patients with Usher Syndrome type II. J Hum Genet. 2009;54: 732-8.
212. Weston MD, Kelley PM, Overbeck LD, Wagenaar M, Orten DJ, Hasson T, et al. Myosin VIIA mutation screening in 189 Usher syndrome type 1 patients. Am J Hum Genet. 1996;59: 1074-83.
213. Watanabe S, Umeki N, Ikebe R, Ikebe M. Impacts of Usher syndrome type IB mutations on human myosin VIIa motor function. Biochemistry. 2008;47: 9505-13.
214. Jaijo T, Aller E, Garcia-Garcia G, Aparisi MJ, Bernal S, Avila-Fernandez A, et al. Microarray-based mutation analysis of 183 Spanish families with Usher syndrome. Invest Ophthalmol Vis Sci. 2010;51: 1311-7.
215. Roux AF, Faugere V, Le Guedard S, Pallares-Ruiz N, Vielle A, Chambert S, et al. Survey of the frequency of USH1 gene mutations in a cohort of Usher patients shows the importance of cadherin 23 and protocadherin 15 genes and establishes a detection rate of above 90%. Journal of Medical Genetics. 2006;43: 763-8.
216. Roux AF, Faugere V, Vache C, Baux D, Besnard T, Leonard S, et al. Four-year follow-up of diagnostic service in USH1 patients. Invest Ophthalmol Vis Sci. 2011;52: 4063-71.
217. Gerber S, Bonneau D, Gilbert B, Munnich A, Dufier JL, Rozet JM, et al. USH1A: chronicle of a slow death. Am J Hum Genet. 2006;78: 357-9.
218. Corton M, Nishiguchi KM, Avila-Fernandez A, Nikopoulos K, Riveiro-Alvarez R, Tatu SD, et al. Exome sequencing of index patients with retinal dystrophies as a tool for molecular diagnosis. PLoS One. 2013;8: e65574.
219. Weston MD, Eudy JD, Fujita S, Yao S, Usami S, Cremers C, et al. Genomic structure and identification of novel mutations in usherin, the gene responsible for Usher syndrome type IIa. Am J Hum Genet. 2000;66: 1199-210.
220. Dreyer B, Brox V, Tranebjaerg L, Rosenberg T, Sadeghi AM, Moller C, et al. Spectrum of USH2A mutations in Scandinavian patients with Usher syndrome type II. Hum Mutat. 2008;29: 451.

221. Cideciyan AV, Swider M, Aleman TS, Sumaroka A, Schwartz SB, Roman MI, et al. ABCA4-associated retinal degenerations spare structure and function of the human parapapillary retina. Invest Ophthalmol Vis Sci. 2005;46: 4739-46.
222. Zwaenepoel I, Verpy E, Blanchard S, Meins M, Apfelstedt-Sylla E, Gal A, et al. Identification of three novel mutations in the USH1C gene and detection of thirty-one polymorphisms used for haplotype analysis. Hum Mutat. 2001;17: 34-41.

Supp. Table S6. Individuals suggested for clinical reevaluation after genomic testing.

study id	referral group	gene reported
12007398	USHER	*ABCA4*
12010090	OTHER – atypical STGD	*BBS5*
12010854	RP/RCD	*CHM*
13012171	RP/RCD	*CHM*
13015421	RP/RCD	*CNGA3*
13004358	RP/RCD	*GPR98*
13017391	ACHM/CD	*GUCY2D*
12002962	RP/RCD	*PCDH15*
13017782	STGD/MD	*RP1*

Supp Table S7. The heterogeneity of inherited retinal disease reported in this study.

causal gene	number of individuals	number of disease referrals	disease referrals
ABCA4	20	5	CD (1), CRD (4), RP/RCD (3), STGD/MD (11), USHER (1)
AIPL1	3	2	EORD/LCA (2), STGD/MD (1)
BBS1	3	1	BBS (3)
BBS10	1	1	BBS (1)
BBS12	1	1	BBS (1)
BBS4	1	1	BBS (1)
BBS5	1	1	OTHER (1)
BBS7	1	1	BBS (1)
BEST1	3	2	RP/RCD (1), STGD/MD (2)
C1QTNF5	1	1	RP/RCD (1)
CDH23	1	1	USHER (1)
CDHR1	1	1	RP/RCD (1)
CEP290	12	4	CRD (1), JOUBERT (1), EORD/LCA (9), RP/RCD (1)
CERKL	15	4	CD (2), CRD (4), RP/RCD (5), STGD/MD (4)
CHM	2	1	RP/RCD (2)
CLRN1	3	2	RP/RCD (2), USHER (1)
CNGA1	2	1	RP/RCD (2)
CNGA3	6	3	CD (4), CRD (1), RP/RCD (1)
CNGB1	4	1	RP/RCD (4)
CNGB3	7	1	CD (7)
CRB1	21	3	CRD (2), EORD/LCA (10), RP/RCD (9)
CRX	4	3	CRD (1), EORD/LCA (1), STGD/MD (2)
DFNB31	1	1	USHER (1)
EYS	7	1	RP/RCD (7)
FAM161A	3	1	RP/RCD (1)
FZD4	2	1	FEVR (2)
GNAT2	1	1	CD (1)
GPR98	1	1	RP/RCD (1)
GUCA1A	1	1	RP/RCD (1)
GUCY2D	11	3	CD (3), EORD/LCA (7), RP/RCD (1)
IMPG2	3	1	RP/RCD (1)
KCNV2	1	1	STGD/MD (1)
LCA5	1	1	EORD/LCA (1)
LRAT	1	1	EORD/LCA (1)
LRP5	1	1	FEVR (1)
MERTK	4	1	RP/RCD (4)
MYO7A	5	2	EORD/LCA (1), USHER (4)
NR2E3	1	1	RP/RCD (1)
NRL	1	1	RP/RCD (1)
OTX2	1	1	RP/RCD (1)
PCDH15	1	1	RP/RCD (1)

PDE6A	1	1	RP/RCD (1)
PDE6B	1	1	RP/RCD (1)
PDE6C	1	1	CD (1)
PROM1	8	3	CRD (3), EORD/LCA (1), RP/RCD (4)
PRPF31	8	2	CRD (1), RP/RCD (7)
PRPF8	4	1	RP/RCD (1)
PRPH2	1	1	STGD/MD (1)
RDH12	4	2	EORD/LCA (3), RP/RCD (1)
RDH5	1	1	OTHER (1)
RHO	6	1	RP/RCD (6)
RP1	10	2	RP/RCD (9), STGD/MD (1)
RP2	5	2	EORD/LCA (1), RP/RCD (4)
RPE65	5	2	EORD/LCA (4), RP/RCD (1)
RPGR	7	2	CRD (1), RP/RCD (6)
RPGRIP1	8	2	CRD (1), EORD/LCA (7)
SNRNP200	5	1	RP/RCD (1)
SPATA7	2	2	EORD/LCA (1), RP/RCD (1)
TIMP3	1	1	RP/RCD (1)
TOPORS	1	1	RP/RCD (1)
USH1C	1	1	USHER (1)
USH2A	31	2	RP/RCD (13), USHER (18)

Supp. Table 8. Variants clinically reported as a novel cause of disease.

hg19 genomic co-ordinate	Variant annotation	Patient Study ID	Referral subtype
missense			
chr1:197325978	CRB1 c.1006T>C p.(Cys336Arg)	13005842	CRD
chr1:197396763	CRB1 c.2308G>A p.(Gly770Ser)*	11000824	RP/RCD
chr1:197398735	CRB1 c.2833G>A p.(Gly945Arg)	13003909	RP/RCD
chr1:197396764	CRB1 c.2309G>A p.(Gly770Asp)*	12014047	RP/RCD
chr1:197404010	CRB1 c.3017C>T p.(Ser1006Phe)*	12014047	RP/RCD
chr1:215847475	USH2A c.13778C>T p.(Ser4593Leu)	12010758	USHER
chr1:215847812	USH2A c.13441A>G p.(Arg4481Gly)	13015666	USHER
chr1:215853690	USH2A c.12095G>T p.(Gly4032Val)	13011433	USHER
chr1:215901725	USH2A c.11713C>T p.(Arg3905Cys)*	13005697	USHER
chr1:215940074	USH2A c.10996T>G p.(Cys3666Gly)	13012581	USHER
chr1:216173784	USH2A c.6446C>A p.(Pro2149Gln)	12008422	USHER
chr1:216221921	USH2A c.6118T>G p.(Cys2040Gly)	13003723	RP/RCD
chr1:216221935	USH2A c.6104G>A p.(Cys2035Tyr)	13008037	RP/RCD
chr1:216373196	USH2A c.3584G>T p.(Cys1195Phe)	12006031	USHER
chr1:94471037	ABCA4 c.6107A>G p.(Tyr2036Cys)	13012619	STGD/MD
chr1:94520688	ABCA4 c.2566T>A p.(Tyr856Asn)	13015690	STGD/MD
chr10:95405761	PDE6C c.1892A>G p.(Glu631Gly)	12003493	CD
chr10:95405763	PDE6C c.1894A>T p.(Arg632Trp)	12003493	CD
chr11:68115344	LRP5 c.121C>T p.(Arg41Trp)	13010229	FEVR
chr11:68153802	LRP5 c.1034T>A p.(Leu345Gln)	13010229	FEVR
chr11:76871263	MYO7A c.1135G>A p.(Gly379Arg)	12000202	USHER
chr11:76903286	MYO7A c.4115T>G p.(Val1372Gly)	10002008	USHER
chr11:86662837	FZD4 c.961G>A p.(Val321Ile)	12006042	FEVR
chr12:56115698	RDH5 c.536A>G p.(Lys179Arg)*	13010232	OTHER
chr12:88534765	CEP290 c.148C>T p.(His50Tyr)	12002965	RP/RCD
chr14:68192807	RDH12 c.383T>G p.(Val128Gly)	12012655	EORD/LCA
chr14:68200524	RDH12 c.910T>C p.(Trp304Arg)	12012655	EORD/LCA
chr15:72103895	NR2E3 c.191G>T p.(Cys64Phe)	13012950	RP/RCD
chr16:57937839	CNGB1 c.2681G>A p.(Arg894His)	13000500	RP/RCD
chr16:57951242	CNGB1 c.2096A>G p.(Asp699Gly)	13000500	RP/RCD
chr16:57935339	CNGB1 c.2893G>A p.(Gly965Ser)	13012959	RP/RCD
chr16:57965773	CNGB1 c.1382C>T p.(Thr461Met)	13012959	RP/RCD
chr17:1554982	PRPF8 c.6470T>A p.(Val2157Glu)	13008349	RP/RCD
chr17:7906745	GUCY2D c.380C>T p.(Pro127Arg)	13005797	EORD/LCA
chr19:54621779	PRPF31 c.121C>G p.(Leu41Val)	12011000	CRD
chr19:54625894	PRPF31 c.341T>A p.(Ile114Asn)	13002431	RP/RCD
chr19:54629963	PRPF31 c.916G>A p.(Asp306Asn)	13010305	RP/RCD
chr2:112761560	MERTK c.1866G>C p.(Lys622Asn)	13001452	RP/RCD
chr2:112777090	MERTK c.2180G>A p.(Arg727Gln)*	13000107	RP/RCD
chr2:112779111	MERTK c.2302G>A p.(Ala768Thr)	13005790	RP/RCD
chr2:96955598	SNRNP200 c.2879C>T p.(Ala960Val)	13007688	RP/RCD
chr2:96959219	SNRNP200 c.1871G>A p.(Arg624Lys)	12012775	RP/RCD
chr2:99013190	CNGA3 c.1557G>A p.(Met519Ile)	13012218	CD
chr2:99013564	CNGA3 c.1931T>C p.(Phe644Ser)	13015421	RP/RCD
chr4:47938964	CNGA1 c.1754T>G p.(Met585Arg)	13009346	RP/RCD
chr4:47942803	CNGA1 c.848A>T p.(Asp283Val)	13009346	RP/RCD
chr5:149263008	PDE6A c.2119C>T p.(Arg707Trp)	13002513	RP/RCD

chr5:149323870	PDE6A c.367G>T p.(Asp123Tyr)	13002513	RP/RCD
chr8:55533647	RP1 c.121T>C p.(Tyr41His)	13017782	STGD/MD
chr8:55534065	RP1 c.539T>G p.(Phe180Cys)	13015513	RP/RCD
chr9:117169063	DFNB31 c.1808G>T p.(Gly603Val)	12015458	USHER
chrX:38163914	RPGR c.908G>A p.(Gly303Glu)	13005635	RP/RCD
nonsense			
chr8:55534803	RP1 c.742C>T p.(Gln248Ter)	13015513	RP/RCD
chr1:197403862	CRB1 c.2869C>T p.(Gln957Ter)	13007873	EORD/LCA
chr1:197446947	CRB1 c.4159G>T p.(Glu1387Ter)	12016026	RP/RCD
chr1:215822102	USH2A c.14350G>T p.(Glu4784Ter)	12012730	USHER
chr1:215955512	USH2A c.10612C>T p.(Arg3538Ter)	10003578	RP/RCD
chr1:216373453	USH2A c.3327C>A p.(Tyr1109Ter)	13009036	USHER
chr1:216420075	USH2A c.2661C>G p.(Tyr887Ter)	13013364	USHER
chr1:94528197	ABCA4 c.1873C>T p.(Gln625Ter)	13012365	STGD/MD
chr10:73565598	CDH23 c.7908C>G p.(Tyr2636Ter)	13000951	USHER
chr11:76885842	MYO7A c.1976C>A p.(Ser659Ter)	13005796	EORD/LCA
chr11:76900513	MYO7A c.3628A>T p.(Lys1210Ter)	12006582	USHER
chr11:76919517	MYO7A c.5899C>T p.(Arg1967Ter)*	12000202	USHER
chr12:88477635	CEP290 c.4801C>T p.(Gln1601Ter)	12006834	EORD/LCA
chr12:88510853	CEP290 c.1781T>A p.(Leu594Ter)	13003353	EORD/LCA
chr14:21786006	RPGRIP1 c.1303A>T p.(Lys435Ter)	13002515	EORD/LCA
chr14:21788314	RPGRIP1 c.1445T>A p.(Leu482Ter)	13002689	EORD/LCA
chr14:21793489	RPGRIP1 c.2314C>T p.(Gln772Ter)	13002689	EORD/LCA
chr16:57998062	CNGB1 c.262C>T p.(Gln88Ter)	12012050	RP/RCD
chr17:7919559	GUCY2D c.3175A>T p.(Arg1059Ter)	083830	EORD/LCA
chr19:54631469	PRPF31 c.967G>T p.(Glu323Ter)	13002499	RP/RCD
chr19:54631496	PRPF31 c.994C>T p.(Gln332Ter)	11005352	RP/RCD
chr19:54631562	PRPF31 c.1060C>T p.(Arg354Ter)*	11011655	RP/RCD
chr2:182521541	CERKL c.193G>T p.(Glu65Ter)	13006449; 12013544; 13011267; 13008350; 13000499	STGD/MD; RP/RCD; CRD; STGD/MD; RP/RCD
chr2:62066675	FAM161A c.1464G>A p.(Trp488Ter)	11006153	RP/RCD
chr2:98996670	CNGA3 c.248G>A p.(Trp83Ter)	13015421	RP/RCD
chr3:100947720	IMPG2 c.3634G>T p.(Glu1212Ter)	12014873	RP/RCD
chr3:150690398	CLRN1 c.98G>A p.(Trp33Ter)	13002679	USHER
chr4:155665951	LRAT c.473G>A p.(Trp158Ter)	12004275	EORD/LCA
chr4:47938971	CNGA1 c.1747C>T p.(Arg583Ter)	13000106	RP/RCD
chr4:619706	PDE6B c.291C>A p.(Tyr97Ter)	12007088	RP/RCD
chr6:65016917	EYS c.6137G>A p.(Trp2046Ter)	12002958	RP/RCD
chr6:66112400	EYS c.1155T>A p.(Cys385Ter)	13013377; 13002044; 13004487	RP/RCD; RP/RCD; RP/RCD
chr8:55538474	RP1 c.2032C>T p.(Gln678Ter)	13004227	RP/RCD
chr8:55539191	RP1 c.2749C>T p.(Gln917Ter)	13007209	RP/RCD
chrX:38164014	RPGR c.808C>T p.(Gln270Ter)	13011161	RP/RCD
out-of-frame indels			
chr4:122749847-122749848	BBS7 c.1712_1713delinsAGA p.(Ser571Ter)	12014789	BBS
chr1:197390570-197390571	CRB1 c.1612_1613insCTTA	13012618	EORD/LCA

chr1:216173760	USH2A c.6470delG	13011433	USHER
chr1:216495311	USH2A c.1558delT	11012656	USHER
chr1:94508984	ABCA4 c.3098delA	082863	CRD
chr10:55582113-55582122	PCDH15 c.5385_5394delTCCTCTTCCT	12002962	RP/RCD
chr12:88487674-88487675	CEP290 c.3181_3182delAT	12004181	EORD/LCA
chr12:88508195-88508196	CEP290 c.2052+1_2052+2delGT*	12009433	EORD/LCA
chr14:21793021	RPGRIP1 c.2007delT	13013813	EORD/LCA
chr14:21794230-21794231	RPGRIP1 c.2608_2609insA*	12010986	CRD
chr14:68193897-68193927	RDH12 c.648_658+20del31	13005791	EORD/LCA
chr17:1565308-1565312	PRPF8 c.3910_3914delAACTC	12002571	RP/RCD
chr19:48342929	CRX c.605delG*	13012308	STGD/MD
chr19:54625953	PRPF31 c.400delG	12005728	RP/RCD
chr2:112725802-112725804	MERTK c.933_935delinsTT	13000107	RP/RCD
chr2:182413512-182413513	CERKL c.1045_1046delAT	12011769; 13015689	RP/RCD; CD
chr3:100962762-100962763	IMPG2 c.2412_2413delTG	12014873	RP/RCD
chr4:15985950	PROM1 c.2309delC	088301	CRD
chr4:15995680	PROM1 c.1697delA	12011770	RP/RCD
chr5:89975426-89975429	GPR98 c.5504_5507delTTCC	13004358	RP/RCD
chr6:65612025-65612026	EYS c.2826_2827delAT	13004716	RP/RCD
chr6:80223010-80223016	LCA5 c.633_639delAGCTAGA	12003872	EORD/LCA
chr8:55537904	RP1 c.1462delG	12011625	RP/RCD
chr8:55538040-55538043	RP1 c.1598_1601delGAAA	12013732	RP/RCD
chrX:38150211-38150211	RPGR c.1572+1delG	13018544	RP/RCD
chrX:46713315	RP2 c.507delT	13011266	RP/RCD
chr1:197313565-197313566	CRB1 c.807dupA	12005771	RP/RCD
chr10:85956379-85956380	CDHR1 c.270dupC	13002976	RP/RCD
chr14:88897545-88897546	SPATA7 c.1058dupC	12012257	EORD/LCA
chr16:57938727-57938728	CNGB1 c.2544dupG	12012050	RP/RCD
chr3:101039148-101039149	IMPG2 c.68dupA	12002961	RP/RCD
canonical splice			
chr1:197403835	CRB1 c.2843-1G>A	12007024	EORD/LCA
chr1:215914880	USH2A c.11549-1G>A	12011781	RP/RCD
chr1:216173905	USH2A c.6326-1G>A	12007911	USHER
chr1:216369894	USH2A c.4251+1G>A	12015030	USHER
chr1:216380775	USH2A c.3158-2A>G	13008753	USHER
chr1:68905008	RPE65 c.726-2A>C	13015227	EORD/LCA
chr10:73461778	CDH23 c.2398-1G>T	13000951	USHER
chr12:88532921	CEP290 c.297+1G>T*	13001467	EORD/LCA
chr14:68196099	RDH12 c.848+2T>C	13005789	EORD/LCA
chr15:73023645	BBS4 c.712-1G>A	12009772	BBS
chr19:48337801	CRX c.100+1G>C	13009064	STGD/MD
chr4:16000112	PROM1 c.1579-1G>C*	13018482; 12009616	RP/RCD; CRD
chr4:16014897	PROM1 c.1141+1G>A	13004232	RP/RCD
chr4:16025917	PROM1 c.694+1G>A	13018482	RP/RCD
synonymous			
chr1:216424273	USH2A c.2139C>T p.(Gly713Gly)	12000462	RP/RCD
chr10:55581921	PCDH15 c.5586C>A p.(Ala1862Ala)	12002962	RP/RCD
chr17:1564976	PRPF8 c.4131C>T p.(Ser1377Ser)	12003878	RP/RCD

chr4:16000058	PROM1 c.1632G>T p.(Gly544Gly)	12011770;<br>13004232	RP/RCD;<br>RP/RCD
splice region			
chr11:17542412	USH1C c.1210+5G>A	12013349	USHER
chr11:76853873	MYO7A c.132+5G>A	12000202	USHER
chr4:16014893	PROM1 c.1141+5G>C	12013927	CRD
chr4:16025914	PROM1 c.694+4A>T	13002979	EORD/LCA
chr9:117170303	DFNB31 c.1627-5T>A	12015458	USHER
in-frame indels			
chrX:46713068	RP2 c.260_268del p.(Thr87_Cys89del)	11011508	EORD/LCA
chr14:24550532	NRL c.586_627dup p.(Ala196_Ala209dup)	13010253	RP/RCD
chr14:88904207-88904218	SPATA7 c.1241_1252del12 p.(Val414_Val417del)	12012257	EORD/LCA

* indicates variants which have been described in the literature since the variant was clinically reported as a novel cause of a disease: *CRB1* c.2308G>A p.(Gly770Ser)[1]; *CRB1* c.2309G>A p.(Gly770Asp)[2]; *CRB1* c.3017C>T p.(Ser1006Phe)[3]; *USH2A* c.11713C>T p.(Arg3905Cys)[4]; *RDH5* c.536A>G p.(Lys179Arg)[5]; *MERTK* c.2180G>A p.(Arg727Gln)[6]; *CEP290* c.297+1G>T[7]; *PROM1* c.1579-1G>C[8]; *MYO7A* c.5899C>T p.(Arg1967Ter)[9]; *PRPF31* c.1060C>T p.(Arg354Ter)[10]; *CEP290* c.2052+1_2052+2delGT[11]; *RPGRIP1* c.2608_2609insA[12]; *CRX* c.605delG[13]

1. Eisenberger T, Neuhaus C, Khan AO, et al. Increasing the Yield in Targeted Next-Generation Sequencing by Implicating CNV Analysis, Non-Coding Exons and the Overall Variant Load: The Example of Retinal Dystrophies. PLoS One 2013;8:18.
2. Corton M, Tatu SD, Avila-Fernandez A, et al. High frequency of CRB1 mutations as cause of Early-Onset Retinal Dystrophies in the Spanish population. Orphanet J Rare Dis 2013;8:20.
3. Li SQ, Shen T, Xiao XS, Guo XM, Zhang QJ. Detection of CRB1 mutations in families with retinal dystrophy through phenotype-oriented mutational screening. Int J Mol Med 2014;33:913-8.
4. Krawitz PM, Schiska D, Krüger U, et al. Screening for single nucleotide variants, small indels and exon deletions with a next-generation sequencing based gene panel approach for Usher syndrome. Molecular Genetics & Genomic Medicine 2014:n/a-n/a.
5. Maranhao B, Biswas P, Duncan JL, et al. exomeSuite: Whole exome sequence variant filtering tool for rapid identification of putative disease causing SNVs/indels. Genomics 2014;103:169-76.
6. Coppieters F, Van Schil K, Bauwens M, et al. Identity-by-descent-guided mutation analysis and exome sequencing in consanguineous families reveals unusual clinical and molecular findings in retinal dystrophy. Genet Med 2014;16:671-80.
7. Wang J, Zhang VW, Feng YM, et al. Dependable and Efficient Clinical Utility of Target Capture-Based Deep Sequencing in Molecular Diagnosis of Retinitis Pigmentosa. Investigative Ophthalmology & Visual Science 2014;55:6213-23.
8. Boulanger-Scemama E, El Shamieh S, Demontant V, et al. Next-generation sequencing applied to a large French cone and cone-rod dystrophy cohort: mutation spectrum and new genotype-phenotype correlation. Orphanet journal of rare diseases 2015;10:85.
9. Shahzad M, Sivakumaran TA, Qaiser TA, et al. Genetic Analysis through OtoSeq of Pakistani Families Segregating Prelingual Hearing Loss. Otolaryngol Head Neck Surg 2013;149:478-87.

10. Sullivan LS, Bowne SJ, Reeves MJ, et al. Prevalence of Mutations in eyeGENE Probands With a Diagnosis of Autosomal Dominant Retinitis Pigmentosa. Investigative Ophthalmology & Visual Science 2013;54:6255-61.
11. Wang X, Wang H, Sun V, et al. Comprehensive molecular diagnosis of 179 Leber congenital amaurosis and juvenile retinitis pigmentosa patients by targeted next generation sequencing. J Med Genet 2013;50:674-88.
12. Fakhratova M. Identification of a Novel LCA6 Mutation in an Emirati Family. Ophthalmic Genet 2013;34:234-7.
13. Hull S, Arno G, Plagnol V, et al. The Phenotypic Variability of Retinal Dystrophies Associated With Mutations in CRX, With Report of a Novel Macular Dystrophy Phenotype. Investigative Ophthalmology & Visual Science 2014;55.
